# Choroid Plexus Fibroblast–ILC2 Niche Promotes Adult Hippocampal Neurogenesis after Traumatic Brain Injury

**DOI:** 10.1002/advs.202415984

**Published:** 2025-07-03

**Authors:** Shiqi Gao, Xiaoming Guo, Sixuan Tian, Huaping Huang, Caidi Ying, Junjie Wang, Jiahao Zhang, Jun Lin, Anwen Shao, Jingyu Wang, Yuan Hong

**Affiliations:** ^1^ Department of Neurosurgery The Second Affiliated Hospital Zhejiang University School of Medicine Hangzhou 310009 China; ^2^ Clinical Research Center for Neurological Diseases of Zhejiang Province Hangzhou 310009 China; ^3^ Zhejiang Key Laboratory of Research and Transformation for Major Neurosurgical Diseases Hangzhou 310009 China; ^4^ Ningbo Municipal Hospital of Traditional Chinese Medicine (TCM) Affiliated Hospital of Zhejiang Chinese Medical University Ningbo 315010 China

**Keywords:** choroid plexus, fibroblast, group 2 innate lymphoid cell, neurogenesis, single‐cell RNA sequencing, traumatic brain injury

## Abstract

Mounting evidence has indicated that immune signals originating from the brain's border tissues will exert a profound influence on brain parenchyma neural cells. However,  the structural component alterations and immune cell infiltration characteristics of choroid plexus (ChP) following traumatic brain injury (TBI) remain incompletely understood. Here, using single‐cell RNA sequencing and histological analysis, the accumulation of group 2 innate lymphoid cell (ILC2) in the ChP stroma post‐TBI is identified. Intracerebroventricular adoptive transfer of ILC2 is further indicated to exhibit a tendency to colonize the ChP and significantly alleviate pathogenic immune infiltration during the acute phase of TBI, as well as maintain hippocampal integrity during the chronic phase. Sensory‐motor function and memory impairments in TBI mice are also improved under ILC2 treatment. Mechanistically, ILC2 is induced by ChP fibroblasts derived IL33 and anchored to stroma fibroblasts via the VCAM‐1/Integrin α4β7 pathway. Furthermore, single‐nucleus RNA sequencing of the hippocampus reveals that ILC2‐derived AREG promotes the initiation of neurogenesis by interacting with EGFR on early‐stage neurogenic cells. Overall, these findings highlight that ChP‐resident ILC2, through optimizing the immune microenvironment and promoting neurogenesis after TBI, may represent a potential therapeutic strategy.

## Graphical abstract

This study investigates the role of group 2 innate lymphoid cells (ILC2) in the choroid plexus (ChP) following traumatic brain injury (TBI). ILC2 accumulation alleviates immune infiltration, preserves hippocampal integrity, and improves sensory‐motor and memory functions. Mechanistically, ILC2, induced by ChP fibroblasts, promotes neurogenesis through AREG‐EGFR interaction, suggesting a potential therapeutic strategy for TBI

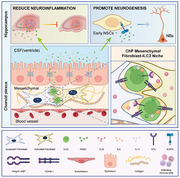

## 1 Introduction

Central nervous system (CNS)‐associated border immunoregulation has been revealed to have profound effects on parenchymal non‐immune CNS cells in both healthy and disease states.^[^ ^1^ ^]^ As an internal “border compartment” within the brain parenchyma, the choroid plexus (ChP) plays a pivotal role in maintaining physiological homeostasis and immune surveillance during disease.^[^ ^2^ ^]^ Studies have highlighted the ChP as a critical pathway for immune cells to infiltrate the brain during infection or injury.^[^ ^3^, ^4^ ^]^ Recent evidence suggests that specific immune cells recruited to the ChP, or signals derived from it, are essential for neural development and neurological recovery.^[^ ^5^, ^6^, ^7^ ^]^ The ChP's abundant blood supply and its unique structure make it an ideal site for the recruitment and residence of immune cells, particularly when the brain microenvironment fluctuates. Therefore, we hypothesize that, during the recovery phase following traumatic brain injury (TBI), certain immune cell types may reside in the ChP and play an important role in neurological recovery by regulating the immune environment to support tissue repair or through other direct mechanisms.

Group 2 innate lymphoid cells (ILC2) are a recently identified class of innate effector cells that function similarly to Th2 cells. These cells are characterized by the expression of markers such as GATA3, ST2, CD127 (IL‐7RA), CD25, ICOS, CD90.2 (Thy1.2), and IL17BR, but lack lymphoid (e.g., CD3, CD19) or myeloid (e.g., CD11b, CD11c, LY6G, LY6C) markers.^[^ ^8^ ^]^ ILC2 are typically found in specific non‐lymphoid tissues, such as the mucosal barriers of the lungs, intestine, uterus, fat, and skin. They respond rapidly to the alteration of factors like IL33, IL25, and TSLP in the microenvironment.^[^ ^9^ ^]^ Type II immune response mediated by ILC2 is one of the deleterious pathogenic mechanisms in various tissue fibrosis, as well as allergic diseases represented by airway hyperresponsiveness.^[^ ^10^, ^11^, ^12^ ^]^ Nevertheless, ILC2 are also committed to immunosuppressive effects and homeostasis maintenance.^[^ ^13^ ^]^ Growing evidence supports their involvement in the repair and remodeling of liver, kidney, gastrointestinal tract, and other tissues, as well as protecting the cardiovascular system from conditions like aneurysms and atherosclerosis.^[^ ^14^, ^15^, ^16^, ^17^ ^]^ These protective effects are primarily mediated by Th2 cytokines secreted by ILC2, represented by IL‐5, IL‐13, and AREG.^[^ ^18^, ^19^ ^]^ Despite these findings, research on ILC2 in the CNS remains shallow.

Limited data suggest that ILC2 are present in the dura mater of both humans and mice, where they contribute to cortical inhibitory synapse maturation and adult social behavior.^[^ ^20^ ^]^ Additionally, dura‐resident ILC2 have been shown to support repair following spinal cord injury.^[^ ^21^ ^]^ Some recent studies indicated that ILC2 are revealed to be augmented in the injured hemisphere during the acute phase of cerebral hemorrhage and ischemic stroke models according to the results of flow cytometry.^[^ ^22^, ^23^ ^]^ It was additionally proved that IL33‐induced amplification of endogenous ILC2 and adoptive transfer homologous ILC2 can reduce immune infiltration and deleterious inflammatory responses during the acute injury phase.^[^ ^22^, ^23^ ^]^ However, histological evidence for the presence of ILC2 in the brain parenchyma in healthy or disease states is still lacking. Notably, ILC2 have been found to accumulate in the ChP of aged mice, where they exhibit greater proliferative and functional activity than meningeal ILC2.^[^ ^24^ ^]^ This accumulated ILC2 is thought to contribute to cognitive decline associated with aging.^[^ ^24^ ^]^  The only study to date examining ILC2 in TBI has shown their presence in both the meninges and cerebrospinal fluid (CSF) of TBI mice and patients with severe brain injury.^[^ ^25^ ^]^ However, it remains unclear whether ILC2 exist in other brain regions and how they might contribute to immune regulation and brain recovery.

In this study, we adopted single‐cell RNA sequencing (scRNA‐seq) to identify the augmentation of ILC2 in the ChP mesenchyme with the association of fibroblasts proliferation during the progression following TBI. Our results revealed that intracerebroventricular (i.c.v.) transplantation of homologous ILC2 significantly inhibited immune infiltration and promoted neurogenesis in the hippocampus of TBI mice. Mechanistically, massive residence of ILC2 in the ChP mesenchyme is collaboratively regulated by the IL33/ST2 and VCAM‐1/integrin α4β7 pathways. Regarding neurogenesis, AREG secreted by ILC2 initiates the neurogenesis process by interacting with EGFR on neurogenic cells during their early stage. These findings highlight the impact of border‐related immunity on neural cells and suggest a potential novel therapeutic strategy for neurological repair after brain injury.

## 2 Results

### 2.1 ILC2 are Present in the ChP of Healthy Mice, and Amplification of Which is Associated with the Proliferation of ChP Fibroblasts after TBI

**Figure 1:**
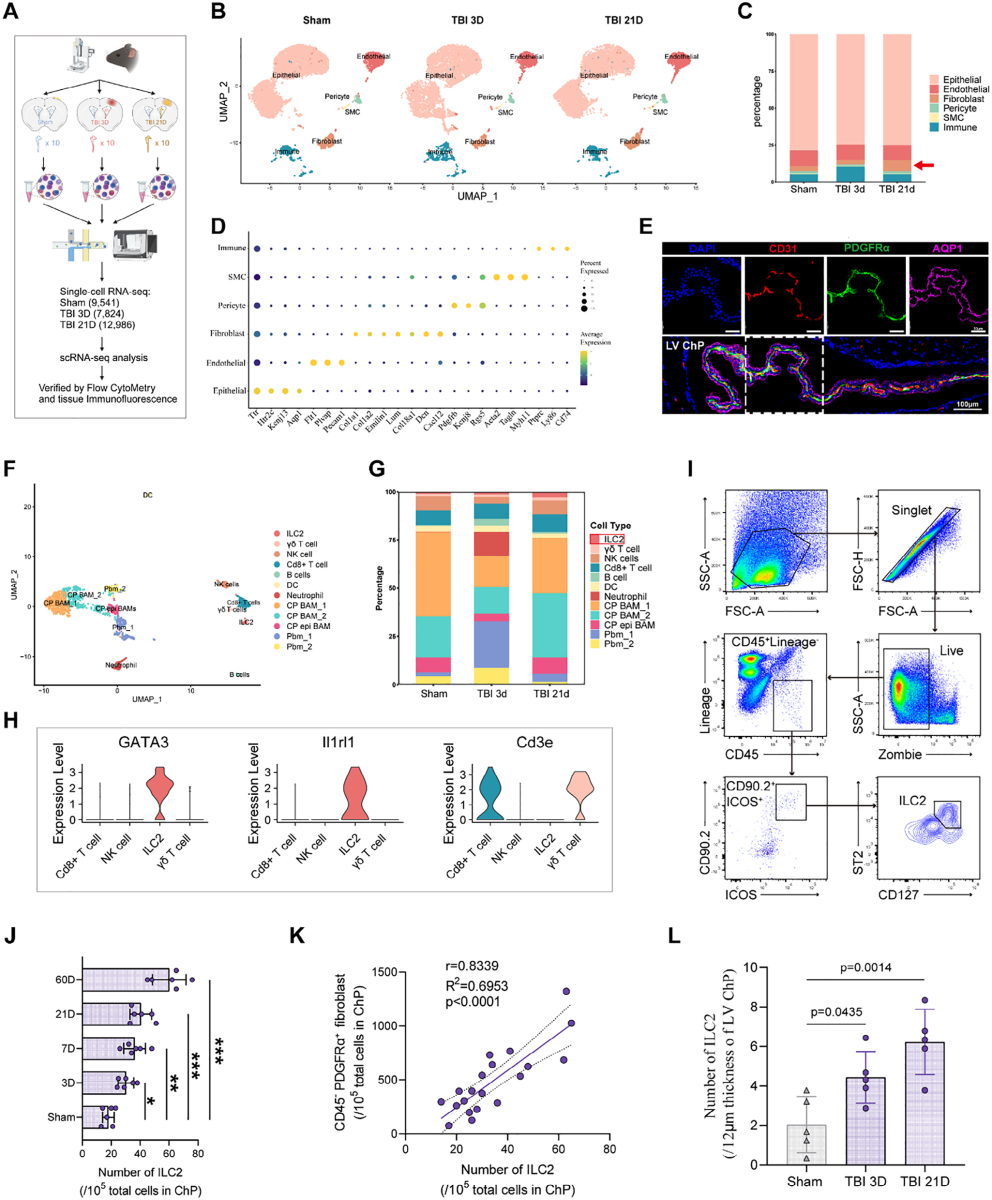
Accumulation of ILC2 in the choroid plexus is associated with the proliferation of fibroblasts after TBI. A) Experimental design for scRNA‐seq and verification methods. n = 10 LV ChP for each scRNA‐seq sample. B) UMAP plots of 6 major cells categories from sham, TBI 3D, TBI 21D groups. C) Stacked bar graph displaying the proportion of each cell cluster among total cells in each group. The red arrow points to fibroblasts. D) Dot plots showing the average expression (color) and the percentage of expressing cells (circle size) for characteristic marker genes (columns) in each cell categories. E) Immunofluorescence markers for major cell category in LV ChP. *Top*: Partial pictures of endothelial (CD31, red), mesenchymal (Col IV, green) and epithelial (AQP1, purple) cells; *bottom*: merged and enlarged picture. F) High‐resolution UMAP plots of 12 sub‐clusters of immune cells from whole cells. G) Stacked bar graph displaying the proportion of each cell cluster among immune cells in each group. H) VlnPlot showing the expression of GATA3, Il1rl1 and Cd3e in 4 clusters of non‐B lymphocytes. I,J) Flow cytometry analysis of Lineage^−^CD90.2^+^ICOS^+^CD127^+^ST2 (Il1rl1)^+^ ILC2 in the ChP after TBI and in sham ChP (per 10^5^ total ChP cells, n = 6). ^*^ *p* < 0.05, ^**^ *p* < 0.01 and ^***^ *p* < 0.001 versus sham group. K) Correlation between the ChP ILC2 and fibroblasts (CD45^−^PDGFRα^+^) frequency. n = 20. L) Immunofluorescence quantification for ILC2 in LV ChP of injured side (related to Figure S1E). n = 5.

The ChP, composed of distinct cell types, is well known to initiate rapid responses to brain injury.^[^ ^3^ ^]^ However, the subacute changes within the ChP following injury are less understood. Dissecting the dynamic changes of ChP structural cells and resident or infiltrating immune cells over time may shed light on specific functions of these cells. We therefore performed scRNA‐seq (10× Genomics) on ChP samples from the lateral ventricle (LV) of the injured hemisphere at 3 days and 21 days after controlled cortical injury (CCI), with a sham group for comparison (**Figure** **1**A). The 3‐day and 21‐day time points were selected to represent the subacute and chronic recovery phases, respectively. Due to the small size of the ChP, we pooled 10 microdissected ChP samples per group for sequencing (Figure 1A). After quality control and batch effect correction, a total of 9541 cells from the sham group, 7824 cells from the 3‐day group, and 12986 cells from the 21‐day group were included in subsequent analyses (Figure 1A).

Based on the ChP structure classification by Neil Dani et al.^[^ ^26^ ^]^, we categorized ChP cells into four major groups: epithelium, endothelium, mesenchyme, and immune cells, with mesenchymal cells consisting of fibroblasts, pericytes, and smooth muscle cells (SMCs). Immunofluorescence staining confirmed the spatial localization of these structural cells in the LV ChP (Figure 1E). Using these classifications and established markers (Figure 1D), we annotated 6 major cell clusters in each group (Figure 1B). The epithelium was the dominant cell type in the ChP, with no significant changes between the subacute and chronic phases. However, a notable increase in immune cell populations was observed at the 3‐day time point (Figure 1B,C). By the 21‐day time point, when the inflammatory response subsided, only fibroblasts showed a twofold increase, while the proportions of other cell types were similar to those in the sham group (Figure 1B,C).

To investigate the immune cell populations in the ChP at different time points, we conducted a higher‐resolution analysis, identifying 12 immune sub‐clusters using classical immune markers (Figure 1F; Figure S1A, Supporting Information). Interestingly, ILC2 were present in both the sham and TBI ChP, although they were relatively scarce. However, their numbers significantly increased during both the subacute and chronic phases following TBI (Figure 1G). Notably, by day 21, ILC2 were the only immune sub‐cluster that was markedly upregulated compared to the sham group (Figure 1G). To confirm the identity of ILC2, we analyzed gene expression for markers such as GATA3, ST2 (Il1rl1), and Cd3e, which are characteristic of ILC2 and distinguish them from other non‐B lymphocytes (Figure 1H; Figure S1C, Supporting Information).

We then monitored the temporal changes of the Lineage^−^CD90.2^+^ICOS^+^CD127^+^ST2^+^ ILC2 population in the ChP using flow cytometry at multiple time points (3, 7, 21, and 60 days) following TBI (Figure 1I). Although ILC2 numbers were slightly elevated during the subacute phase (3 and 7 days), they expanded significantly during the chronic phase (after 21 days) (Figure 1J; Figure S1D, Supporting Information). The scRNA‐seq data indicated a similar expansion pattern between ILC2 and fibroblasts in the ChP (Figure S1B, Supporting Information). Flow cytometry analysis further confirmed that the changes in the populations of ILC2 and fibroblasts were highly correlated (Figure 1K). Additionally, we used immunofluorescence staining with CD45^+^, GATA3^+,^ and CD3^−^ markers to provide further evidence of ILC2 presence in the ChP (Figure S1E, Supporting Information). Although ILC2 were not observed in every slice (12 µm thickness) of the LV ChP by immunofluorescence, we quantified the average number of LV ChP ILC2 per slice for comparison. The results demonstrated that ILC2 numbers were significantly increased at 3 days post‐TBI compared to the sham group, with a further elevation observed at 21 days post‐TBI. This upregulation pattern was generally consistent with findings from both flow cytometry and scRNA‐seq (Figure S1E, Supporting Information; Figure 1L).

### 2.2 Fibroblasts Constitute the Major Source of IL33 in the ChP, and the Protein Levels of IL33 Significantly Upregulated in the ChP after TBI

**Figure 2:**
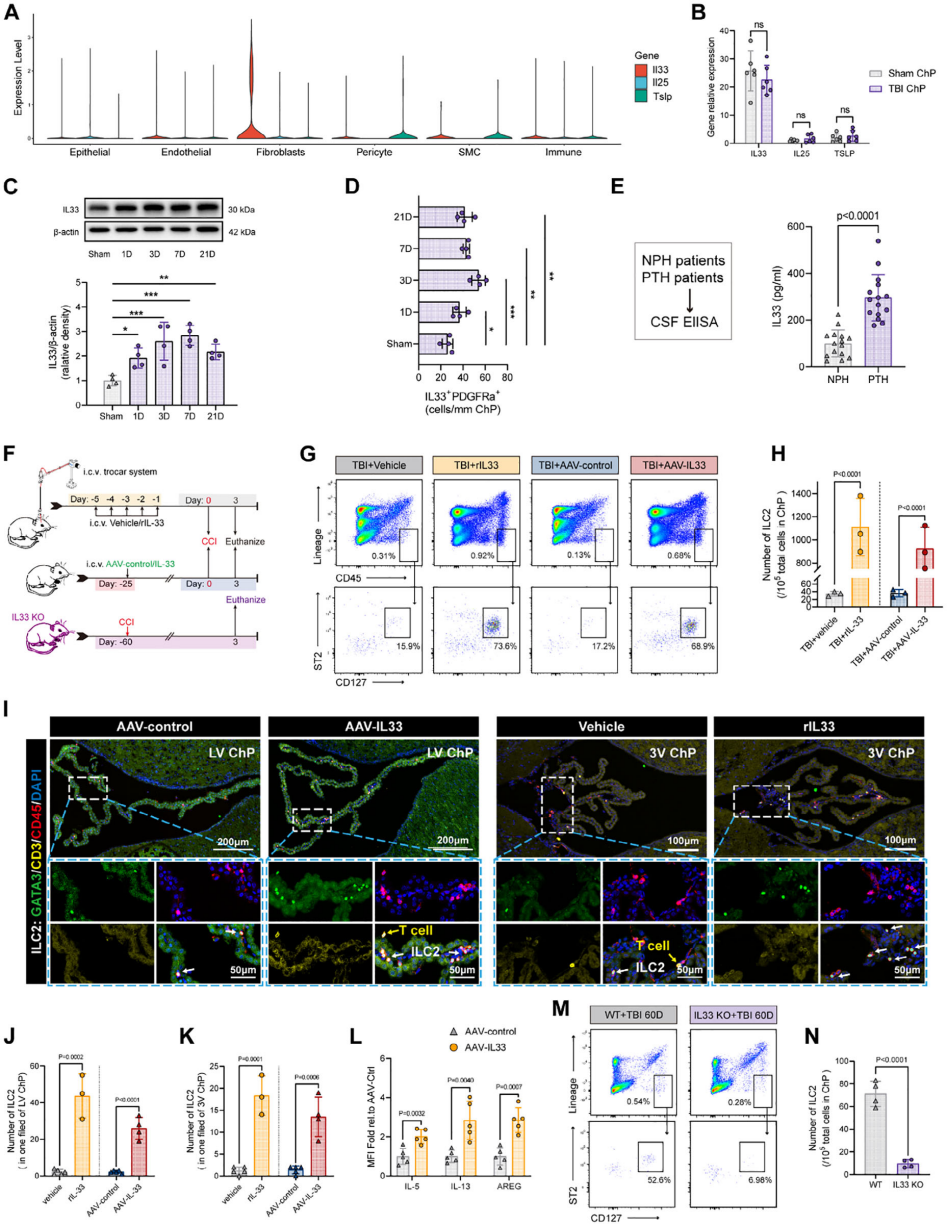
ChP fibroblasts secreted IL33 that specifically induced the aggregation of ILC2 in ChP. A) VlnPlot showing the expression of IL33, IL25 and TSLP in epithelial, endothelial, mesenchymal (fibroblast, pericyte and SMC), and immune cells. B) Relative mRNA expression of IL33, IL25 and TSLP in the ChP from sham and TBI mice. n = 6. C) Representative western blot images and densitometric quantification of IL33 in ChP after 1D, 3D, 7D and 21D of TBI and in ChP of sham brain. ^*^ *p* < 0.05, ^**^ *p* < 0.01 and ^***^ *p* < 0.001 versus sham group, n = 4. D) Quantitative analysis of IL33 relative density in ChP after 1D, 3D, 7D and 21D of TBI and in ChP of sham brain, related to immunofluorescence images of Figure S2A. ^*^ *p* < 0.05, ^**^ *p* < 0.01 and ^***^ *p* < 0.001 versus sham group, n = 4. (E) Quantification of IL33 protein level in CSF samples from NPH and PTH patients respectively by ELISA assay. n = 15. F) Experimental design for Il33 intraventricular delivery and following workflow. G,H) Representative flow cytometry pseudocolor images and quantification for ILC2 in ChP. n = 3, each sample was merged from 2 mice. I–K) Representative immunofluorescence images and quantification for ILC2 in LV ChP and 3 V ChP. L) MFI fold change of IL5, IL13 and AREG of ILC2 in ChP after treatment of AAV‐IL33 or AAV‐Control. n = 5. M,N) Representative Flow cytometry pseudocolor images and quantification for ILC2 in ChP from WT or IL33 KO mice 60 days after TBI. n = 3, each sample was merged from 3 mice.

ILC2 proliferation and activation are regulated by specific ligands, including IL33, IL25, and TSLP, which bind to receptors on the surface of ILC2.^[^ ^19^ ^]^ To gain insight into the cytokines promoting ILC2 activation and expansion during TBI, we first analyzed the scRNA‐seq data. Our results revealed that IL25 and TSLP were scarcely expressed in any of the six major ChP cell clusters, while IL33 was prominently expressed by fibroblasts (**Figure** **2**A). qRT‐PCR further confirmed that IL25 and TSLP levels were very low in the ChP compared to IL33 (Figure 2B). Notably, the mRNA levels of IL33 did not change significantly after TBI.

Next, we examined the protein expression of IL33 in the ChP after TBI using Western blotting (WB). The results demonstrated that IL33 was upregulated in the ChP during both the subacute and chronic phases following TBI, with the most significant upregulation observed at the 3‐day and 7‐day time points (Figure 2C). The discrepancy between mRNA and protein expression levels of IL33 is consistent with previous reports, as IL33 is stored in the nucleus as an alarm molecule and can be rapidly released in response to injury without the need for transcriptional activation.^[^ ^27^ ^]^ Immunofluorescence co‐localization of IL33 with PDGFRα confirmed that fibroblasts were the major source of IL33 in the ChP (Figure S2A, Supporting Information), as well as verified the protein level upregulation pattern of IL‐33 in ChP after TBI revealed by WB(Figure S2A, Supporting Information; Figure 2D).

Additionally, we collected CSF from 15 patients with posttraumatic hydrocephalus (PTH) and from patients with normal pressure hydrocephalus (NPH) as a control group. ELISA analysis showed that IL33 levels were significantly higher in the CSF of PTH patients compared to NPH patients (Figure 2E).

### 2.3 Overexpression of IL33 in ChP and Daily Injection of rIL33 Into the Ventricles Induce a Considerable Expansion of ILC2 in ChP, While IL33 Knockout Abolishes the Accumulation of ILC2 in ChP During the Chronic Phase of TBI

Having identified IL33 as the key signal for promoting ILC2 expansion in the ChP, we employed several gene regulation strategies to validate its role. As ChP is entirely “immersed” in CSF, maintaining a certain concentration of IL33 in CSF is the most direct and efficient way. To achieve this, we developed a specialized intracerebrventricular injection system (i.c.v. trocar) that allows for repeated injections of recombinant IL33 (rIL33) over five consecutive days with minimal disruption to the mice (Figure 2F). However, this approach does not specifically target the IL33 derived from “ChP fibroblasts.” To address this, we additionally employed an adeno‐associated virus (AAV) vector, AAV‐2/5, which specifically infects ChP tissue^[^ ^5^, ^28^ ^]^ to induce IL33 overexpression locally (Figure 2F). The EGFP fluorescence signal carried by AAV‐2/5 confirmed that the virus specifically targeted the ChP, with no significant infection in other brain regions, apart from the needle tract (Figure S2B, Supporting Information).

To assess the impact of IL33 on ILC2 expansion, we performed flow cytometry on ChP tissue microdissected from the LV, third ventricle (3 V), and fourth ventricle (4 V), and pooled the samples to ensure sufficient cell quantity and activity. The results revealed that both rIL33 injection and AAV‐IL33 infection caused more than 200‐fold expansion of ILC2 in the ChP compared to the vehicle and AAV‐control groups (Figure 2G,H).

Immunofluorescence staining further confirmed these findings, showing substantial ILC2 accumulation in both the lamellar ChP of the LV and clustered ChP of the 3 V after IL33 induction (Figure 2I–K). In this study, we focused on the ChP of the LV and 3 V because of their close anatomical relationship with the hippocampus.^[^ ^29^ ^]^ These ChP are positioned adjacent to each other, effectively forming a boundary that surrounds the hippocampus. This structural arrangement provides a strong anatomical basis for the regulation of the hippocampus through signals from the ChP. Two important distribution patterns of ILC2 in the 3 V ChP were observed: 1) ILC2 were primarily localized at the ChP basement, where fibroblasts and IL33 are also enriched (Figure S2A,I, Supporting Information); and 2) nearly all CD3^−^ ILC2 were confined to the mesenchyme between the epithelial layers, while CD3^+^ T cells were predominantly located outside the epithelium (Figure 2I). Furthermore, in the AAV‐IL33 group, ILC2 expressed higher levels of effector molecules than AAV‐control group, including IL‐5, IL‐13, and AREG, indicating that IL33‐induced ILC2 activation (Figure 2L). In contrast, ILC2 were scarcely detected in the ChP of IL33 knockout mice 60 days after TBI, a time point when significant ILC2 enrichment should have occurred (Figure 2M,N).

### 2.4 ILC2 Are Nearly Absent in the Brain Parenchyma, Even Post TBI or IL‐33 Induction

The presence of ILC2 in brain tissue under both healthy and pathological conditions remains a subject of debate.^[^ ^30^ ^]^ To address this, we collected brain tissue from sham groups and from mice 3, 7, and 21 days post‐TBI. After microdissection to remove the ChP, single‐cell suspensions were prepared for flow cytometry analysis. Our findings revealed that ST2 and CD127 double‐positive ILC2 were sporadically present in flow cytometry scatter plots for both sham and TBI groups, but these instances were negligible and could be disregarded (Figure S2C,D, Supporting Information). We further examined the quantity of ILC2 in the brain parenchyma after continuous intraventricular injection of rIL33 and AAV‐IL33 infection of the ChP. We observed that IL33 signaling contributed, to some extent, to the amplification of ILC2 within the parenchyma under both sham and TBI conditions (Figure S2E,F, Supporting Information). However, the absolute number of ILC2 in the brain parenchyma remained limited compared to the substantial accumulation seen in the ChP (Figure 2G,H; Figure S2E,F, Supporting Information). We speculated that the occasional ILC2 signals detected by flow cytometry might be due to false‐positive detection or contamination from ILC2 at the periventricular ChP‐border zone, which may not have been fully removed during tissue dissection. Our subsequent histological evidence confirmed this hypothesis: no GATA3^+^ ILC2 signals were observed in either the peripheral or core regions of the injury after TBI, even following AAV‐IL33 infection (Figure S2G,H, Supporting Information). Notably, we detected abundant ILC2 accumulation at the basal part of the ChP, specifically at the intersection between the ChP and brain parenchyma (Figure S2G,H, Supporting Information). Due to the close anatomical connection between the base of the ChP and the brain tissue boundary, it is challenging to completely separate the ChP. Therefore, it is anatomically plausible that ILC2 signals observed in brain tissue suspensions may result from contamination by the ChP.

### 2.5 Fibroblasts Anchor ILC2 to the Mesenchymal Niche of ChP via the VCAM‐1/ Integrin α4β7 Interaction

**Figure 3:**
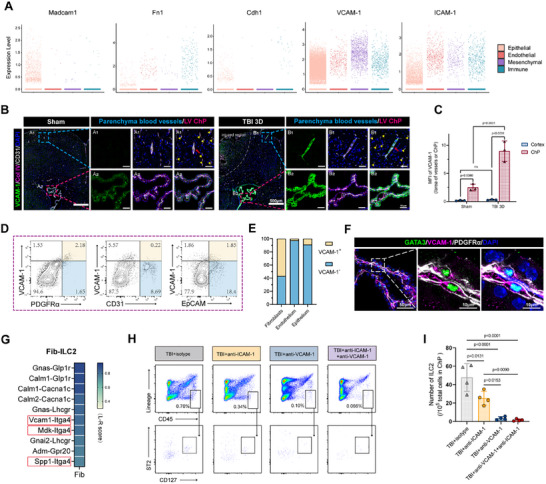
Fibroblasts anchor ILC2 to the mesenchymal niche of ChP via the VCAM‐1/ Integrin α4β7 interaction. A) Expression mode of Madcam1, Fn1, Cdh1, Vcam‐1 and ICAM‐1 in clusters of ChP epithelial, endothelial, mesenchymal and immune cells. B,C) Immunofluorescence staining showing the expression pattern and intensity changes of VCAM‐1 in ChP (red dashed frame) and brain parenchyma (blue dashed frame) after TBI. n = 3. D,E) Representative flow cytometry pseudocolor images and proportions of VCAM‐1 expression in fibroblasts (PDGFRα^+^), endothelial (CD31^+^) and epithelial (EpCAM^+^) from the naïve mouse ChP. F) Immunofluorescence staining showing that VCAM‐1^+^ (purple) and PDGFRα^+^ (white) fibroblasts provide a niche for GATA3^+^ (green) ILC2 on control ChP. G) Heatmap showing the top 10 ligand‐receptor pairs for ILC2 interacting with fibroblasts, assessed by calculating the ligand‒receptor score (L‐R score). The red frame highlights the key ligand‐receptor pairs. H,I) Representative flow cytometry pseudocolor images and quantification for ILC2 in ChP treated with isotype or neutralizing antibodies post TBI. n = 4, each sample was merged from 3 mice.

The unique spatial distribution of ILC2 in the ChP suggests a crucial interaction between ILC2 and the mesenchymal components of the ChP. We hypothesized that a specific molecular mechanism governs the localization of ILC2 in this mesenchymal niche. Immune cell recruitment and tissue localization are heavily regulated by adhesion molecules, which are known to be more abundantly expressed in the ChP compared to other brain regions.^[^ ^3^, ^4^ ^]^ These molecules also respond more rapidly during disease states.^[^ ^4^ ^]^ To identify the ligands in the ChP that may facilitate ILC2 adhesion, we analyzed our scRNA‐seq data. Madcam1, Fn1 fibronectin (Fn1), and Cdh1 (E‐cadherin) were found to be absent in the mesenchyme of ChP, whereas VCAM‐1 and ICAM‐1 are both abundantly expressed (**Figure** **3**A). Notably, VCAM‐1 showed higher relative expression in the mesenchyme compared to other ChP components (Figure 3A), suggesting its specificity in the stroma. In contrast, ICAM‐1 was more prevalent in the ChP endothelium (Figure 3A). These findings led us to focus on VCAM‐1 and ICAM‐1 for further investigation.

The immunofluorescence results indicated that the fluorescence intensity of VCAM‐1 and ICAM‐1 in ChP were significantly higher than that in the vascular units of the brain parenchyma, and both were dramatically upregulated after TBI (Figure 3B,C; Figure S3A,B, Supporting Information). ICAM‐1 can also be detected in the blood vessels of the brain parenchyma in the sham group with an augmentation after injury (Figure S3A,B, Supporting Information). However, for VCAM‐1, its signal in cerebral parenchymal blood vessels is almost absent, only sporadically appearing in some large blood vessels (Figure 3B). The uniqueness of this expression may determine the special distribution of specific immune cells in the ChP. In addition, the results of immunofluorescence also showed the coverage of VCAM‐1 in the mesenchymal components marked by Col IV, while ICAM‐1 showed more epithelial tendency (Figure 3B; Figure S3A, Supporting Information).

The results of flow cytometry also indicated that in contrast to endothelium (CD31^+^) and epithelium (EpCAM^+^), the expression of VCAM‐1 in fibroblasts (PDGFRα^+^) demonstrates high specificity (Figure 3D,E), whereas that of ICAM‐1 does not (Figure S3C,D, Supporting Information). Fibroblasts becoming the structural basis for ILC2 colonization in ChP is feasible in theory. We subsequently confirmed this “niche” structure: PDGFRα^+^VCAM‐1^+^ fibroblast surrounding GATA3^+^ ILC2 in ChP stroma (Figure 3F).

We then conducted two types of cell–cell interaction analyses to further investigate the receptor‐ligand interactions between ILC2 and fibroblasts. CellPhoneDB analysis predicted the “VCAM1‐integrin α4β7 complex” as the most powerful (top 1) ligand‐receptor pair, particularly prominent in both the sham and 21‐day TBI groups (Figure S3E, Supporting Information). Integrin α4β7 is a heterodimeric receptor composed of two subunits, Itga4 and Itgb7.^[^ ^31^ ^]^ The ligand‐receptor (L‐R) score analysis identified VCAM1‐Itga4 as the top 6 interaction between ILC2 and fibroblasts (Figure 3G). It is worth pointing out that the ligand‐receptor pairs listed preceding VCAM1‐Itga4 mainly involve oncogenes, such as GNAS, and calmodulin (CALM), which regulate signaling pathways related to cell division and calcium regulation,^[^ ^32^, ^33^ ^]^ rather than the cell adhesion effects we focus on. Furthermore, these interactions also rank among the top 5 interactions in other ChP cell types (Figure S3F, Supporting Information), suggesting that they are fundamental for maintaining basic cellular functions.

To assess the role of VCAM‐1 in ILC2 expansion, we administered neutralizing antibodies against VCAM‐1 and ICAM‐1, both individually and in combination, into the ventricles of mice subjected to TBI. Flow cytometry analysis revealed that anti‐VCAM‐1 alone, as well as its combination with anti‐ICAM‐1, nearly completely eliminated ILC2 in the ChP post‐TBI, whereas anti‐ICAM‐1 alone had a minimal effect (Figure 3H,I). Additionally, we evaluated the role of these two adhesion molecules in the rIL33‐induced massive accumulation of ILC2 in ChP. Results showed that VCAM‐1 inhibition significantly reduced ILC2 expansion, while ICAM‐1 inhibition also had some effect, though it was less pronounced than VCAM‐1 inhibition (Figure S3G,H, Supporting Information). The reduced clearance efficiency of this neutralizing antibody in the case of large ILC2 aggregation may result from either insufficient antibody titer or the involvement of other adhesion molecules under IL33 induction. However, it is clear that VCAM‐1, expressed by fibroblasts, has a relatively high priority in the attachment of ILC2 to the ChP.

### 2.6 Ventricular Transplantation of Activated ILC2 Specifically Colonize in the ChP, which Reduce Immune Infiltration During the Acute Phase of TBI and Improve Neurological Injury

**Figure 4:**
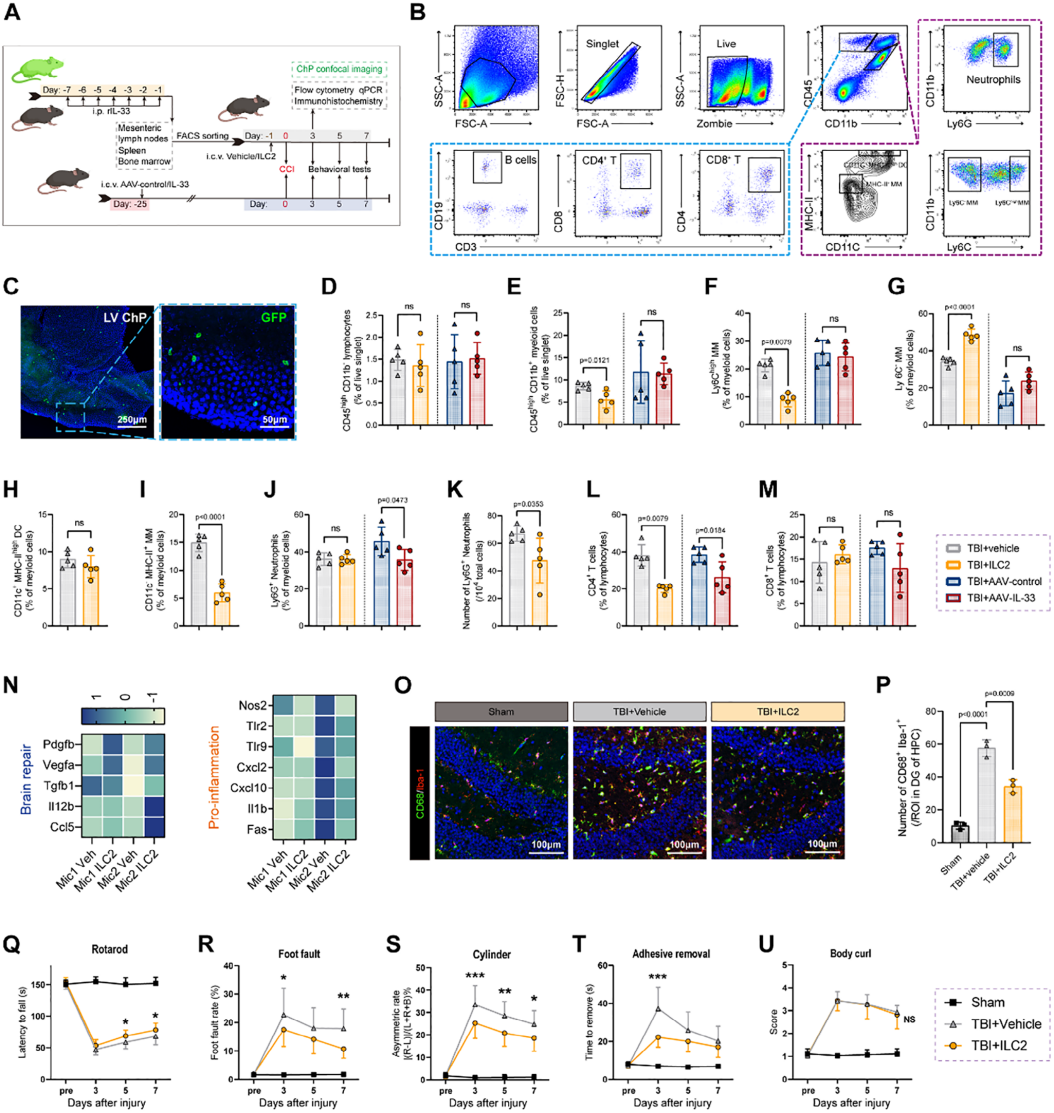
Ventricular transplantation of activated ILC2 specifically colonized in ChP and reduce immune infiltration during the acute phase of TBI and improve neurological injury. A) Experimental design for the FACS sorting and i.c.v. reinfusion of GFP‐labeled ILC2. B) Gating strategy for the flow‐cytometric identification of infiltrated immune cells in the injured hemisphere. C) Representative immunofluorescence images showing the colonization of GFP‐labeled ILC2 in ChP of WT mice. D–M) Proportion or number change of infiltrated immune cells in the injured hemisphere: CD45^high^CD11b^−^ lymphocyte (D), CD45^high^CD11b^+^ myeloid cells (E), LY6C^high^ MM (F), LY6C^−^ MM (G), CD11c^+^MHC‐II^high^ DC (H), CD11c^−^MHC‐11^+^ MM (I), CD11b^+^Ly6G^+^ neutrophil (J, K), CD4^+^ T cells (L), CD8^+^ T cells (M). N) Heatmap showing upregulation of brain repair‐related genes and downregulation of pro‐inflammatory genes in 2 clusters of injured hemisphere microglia treated with ILC2 5 day after TBI. O,P) Quantitative analysis of activated microglia (CD68+Iba‐1+) within a defined region of interest (ROI) in the DG of the hippocampus (HPC). n = 3. (Q‐U) Sensorimotor functions 3 day after TBI were assessed in vehicle and ILC2 treated mice. rotarod test Q), foot fault test R), cylinder test S), adhesive removal test T), body curl score U).

We next sought to investigate the effects of ILC2 on impaired brain tissue and neurological function following TBI. Besides employing AAV‐IL33 to induce the expansion of ILC2 within ChP, we also utilized i.c.v. transplantation of *vivo*‐expanded and activated ILC2 to exclude the direct effect of IL33. Based on previous studies, GFP whole‐body labeling and WT mice achieved a tremendous expansion of ILC2 through intraperitoneal (i.p.) injection of 400 ng IL33 for 7 consecutive days. We selected spleens, mesenteric lymph nodes, and bone marrow with high ILC2 yield for FACS sorting. After combined stimulation with IL2, IL7, and IL33, 2 × 10^5^ ILC2 were transplanted into each mouse via i.c.v. injection (**Figure** **4**A). GFP labeling allowed us to track the specific location of ILC2 in the CNS. GFP fluorescence signals were densely observed in the LV ChP tissue but were absent in the brain parenchyma (Figure 4C). This finding confirmed that ILC2 preferentially localize to the ChP in the CNS and that the subsequent effects of i.c.v. ILC2 injection are likely due to signal changes in the ChP rather than direct effects in the injured region.

To assess the impact of ILC2 on inflammation after TBI, we established a flow cytometry panel to detect various immune cell types in the injured hemisphere 3 days post‐TBI, including CD45^high^CD11b^−^ lymphocyte, CD45^high^CD11b^+^ myeloid cell, LY6C^high^ monocytes/macrophages (MM), LY6C^−^ MM, CD11c^+^MHC‐II^high^ dendritic cells (DC), CD11c^−^MHC‐11^+^ MM, CD11b^+^Ly6G^+^ neutrophil, CD8^+^T cells, CD4^+^ T cells (Figure 4B). Both AAV‐IL33‐induced ILC2 expansion and adoptive transfer of ILC2 did not significantly alter the overall proportions of infiltrating lymphoid cells (Figure 4D). However, compared to AAV‐IL33 induction, adoptive transfer of ILC2 exhibited a superior immunosuppressive effect, reducing the total number of myeloid cells (Figure 4E), pro‐inflammatory MM (LY 6C^+^) (Figure 4F), antigen‐presenting MM (MHC II^+^) (Figure 4I) and neutrophils (Figure 4J,K), while increasing the proportion of anti‐inflammatory MM (LY 6C^−^) (Figure 4G). Furthermore, i.c.v. ILC2 injection also effectively confined the infiltration of CD4^+^ T cells (Figure 4L), although DC cells and CD8^+^ T cells were not remarkably affected (Figure 4H,M). This may be attributed to the negative immune regulation mediated by ILC2.

In addition, we observed a very significant suppression of neutrophils and CD8^+^ T cells in the ChP of mice that were adoptively transferred with ILC2 (Figure S4A,B, Supporting Information). On the contrary, CD4^+^ T cells were difficult to observe by histological means in the ChP post‐TBI, regardless of whether ILC2 was adopted or not. This is consistent with our single‐cell data, which showed no clear subcluster of CD4^+^ T cells in either the sham or TBI groups.

The reduction in inflammation associated with AAV‐IL33 infection may be confounded by IL33's direct effects. Our results showed that IL33 overexpression more effectively reduced neutrophil infiltration after TBI than ILC2 treatment (Figure 4J). The proportion of CD4^+^ T cells and CD8^+^ T cells (no statistical significance) both decreased in AAV‐IL33 treated group compared to control group (Figure 4L,M).

We additionally carried out the previously described FACS panel in the TBI setting of IL33 KO and WT mice. As expected, the immune regulatory effects observed in IL‐33 KO mice were approximately opposite to those associated with IL‐33 overexpression. Specifically, compared to WT mice, IL‐33 KO mice exhibited increased Ly6C^high^ MM, neutrophils, CD8^+^ T cells infiltration (Figure S5C,E,G, Supporting Information), and a significant reduction in Ly6C^−^ MM numbers within the brain parenchyma following TBI (Figure S5D, Supporting Information). The proportions of total lymphocytes, myeloid cells, CD4^+^ T cells, B cells, and resident microglia remained unchanged (Figure S5A,B,F,H,I, Supporting Information).

Infiltration of neutrophils and pro‐inflammatory MM as well as excess T cells are well‐established exacerbating factors after TBI. These data suggest crucial roles of ILC2 cells in resistance to excessive inflammation after TBI. To better bring the protective effect of ILC2 to light, we solely adopted the treatment mode of adoptive transfer of ILC2 for the subsequent research. We analyzed hippocampal snRNA‐seq data collected 6 days post‐TBI (main data shown in Figure 6). The results showed that in the two microglial clusters, genes associated with brain repair (e.g., *Pdgfb*, *Vegfa*, *Tgfb1*, *Il12b*, *Ccl5*) were upregulated, while pro‐inflammatory genes (e.g., *Nos2*, *Tlr9*, *Cxcl2*, *Il1b*, *Fas*) were downregulated in the ILC2 transplantation group (Figure 4N). In addition, microglia activation (CD68^+^Iba‐1^+^) in the hippocampal dentate gyrus (DG) and premotor cortex (PMC) were significantly reduced following ILC2 transfer (Figure 4O,P; Figure S4D,E, Supporting Information). These results indicate that although the quantity of microglia, which account for the majority of the brain parenchyma, did not change under ILC2 treatment, its polarization type has been altered toward an anti‐inflammatory and reparative phenotype.

We also assessed the neurological function of mice during the subacute phase (before 7 days) post‐TBI. The cylinder and adhesive removal tests showed that ILC2 transfer improved sensory function (Figure 4S,T). Motor function, as measured by the rotarod and foot fault tests, also showed some improvement (Figure 4Q,R), though the body curl score remained unchanged (Figure 4U).

### 2.7 The Application of CD90.2 Effectively Eliminates ILC2 in ChP following TBI, Exacerbating Inflammatory Infiltration and Neurological Damage During the Acute Phase

**Figure 5:**
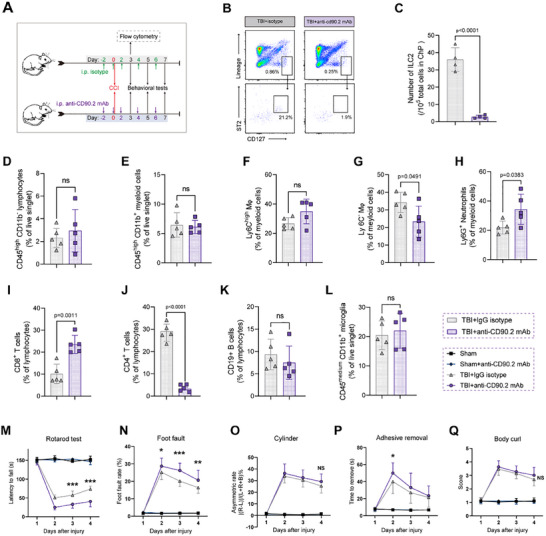
Eliminating ILC2 by applying anti‐CD90.2 neutralizing antibodies exacerbated immune cell infiltration and neurological function damage during the acute phase of TBI. Related to Figure 4. A) Experimental design for the anti‐CD90.2 mAb treatment and FACS testing. B,C) Representative flow cytometry pseudocolor images and quantification for ILC2 in ChP 3 day post TBI. n = 3, each sample was merged from 3 mice. D–L) Proportion change of infiltrated immune cells in injured hemisphere from IgG isotype and anti‐cd90.2 mAb treated group: CD45^high^CD11b^−^ lymphocyte (D), CD45^high^CD11b^+^ myeloid cell (E), LY6C^high^ MM (F), LY6C^−^ MM (G), Ly6G^+^ neutrophil (H), CD8^+^ T cell (I), CD4^+^ T cell (J), CD19^+^ B cell (K), CD11b^+^CD45^media^ microglia (L). M–Q) Sensorimotor functions 3 days after TBI were assessed in IgG isotype and anti‐cd90.2 mAb treated mice. rotarod test (M), foot fault test (N), cylinder test (O), adhesive removal test (P), body curl score (Q).

To further assess the necessity of ILC2 for neuroprotection, we attempted to eliminate ILC2 using i.p. injections of anti‐CD90.2 mAb every other day, as previously reported.^[^ ^23^, ^34^ ^]^ Specifically, anti‐CD90.2 mAb was i.p. injected at a dose of 300 µg every two days until sacrificed as previously described (**Figure** **5**A). A previous study pointed out that this method could not achieve the elimination of ILC2 in the dura mater,^[^ ^21^ ^]^ but our data showed that the administration of anti‐CD90.2 mAb effectively inhibited the accumulation of ILC2 in ChP (Figure 5B,C). Results indicated that anti‐CD90.2 mAb inevitably decreased the proportion of CD4^+^ T cells when eliminating ILC2 (Figure 5J). It may be because of the direct inhibition of T cell subtypes that express CD90.2, such as helper T cells or regulatory T cells.^[^ ^35^ ^]^ Notably, anti‐CD90.2 mAb treatment exacerbated neutrophil infiltration (Figure 5H), LY 6C^high^MM (Figure 5F), and CD8^+^ T cells (Figure 5I) in the traumatic hemisphere, while reducing the proportion of LY 6C^−^ MM (Figure 5G). The proportion of total lymphocytes, myeloid cells, B cells, and resident microglia did not change significantly (Figure 5D,E,K,L). Behavioral testing revealed that the elimination of ILC2 by anti‐CD90.2 mAb aggravated motor deficits, as evidenced by shorter latencies to fall in the rotarod test and increased foot fault rates (Figure 5M,N).  Sensory function also worsened, as indicated by longer adhesive removal times on day 2 post‐TBI in the anti‐CD90.2 mAb group compared to the IgG isotype group (Figure 5P). The results for body curl and cylinder did not change significantly (Figure 5O,Q).

### 2.8 Adoptive Transfer of ILC2 Significantly Promotes Hippocampal Repair and Improves Memory Impairment in the Long‐term After TBI, Which May be Mediated by AREG‐dependent Neurogenesis Acceleration

**Figure 6:**
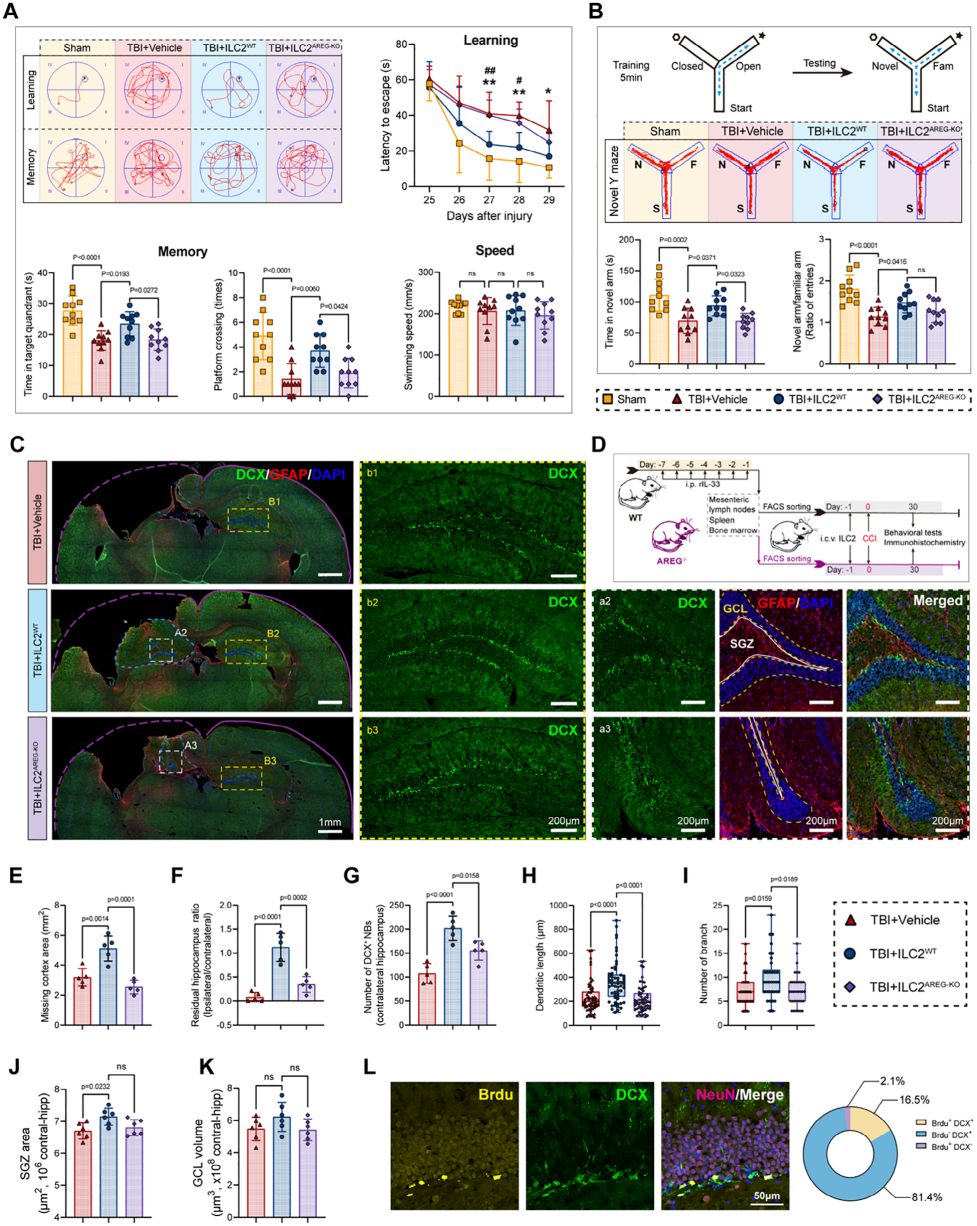
Ventricular transplantation of ILC2 significantly promotes neurogenesis in the hippocampus, improve hippocampal repair and long‐term memory damage after TBI, while ILC2 isolated from AREG^−/−^ mice lakes this repairing effect. A) Spatial learning and spatial memory functions were evaluated by Morris water maze at 25–30 days after injury. The swimming speed of mice was also recorded. Representative images showing the swimming paths of mice during training and testing. n = 10. B) Cognitive functions were additionally evaluated in a new “Y maze”. Experimental schematic diagram and representative images showing the running paths during testing. n = 10. C,E–K) Immunofluorescence images showing the quantification (G) and morphology (H, I) of neuroblasts (DCX+, green), as well as the degree of the defect and morphological changes of the cerebral cortex and hippocampus (E, F) in the coronal section of the brain from vehicle, ILC2WT and ILC2AREG‐KO treated TBI mice. The purple and blue lines indicate the cortical contour and residual hippocampus contour respectively; The DG region of injured and contralateral hippocampus enclosed by the white and yellow box respectively in the left image were enlarged in the middle and right image; The yellow dashed line and white solid line in right picture indicate the deformed granule cell layer (GCL) region and subgranular zone (SGZ) area of the hippocampal DG region respectively. n = 5 for panels E‐G, n = 60 for panel H and n = 35 for panel I. (B) Experimental design for the FACS sorting strategies and following workflow. (J, K) Quantification for SGZ area (J) and GCL volume (K) of contralateral hippocampus. n = 6. L) Immunofluorescence images indicating BrdU (yellow) and DCX (green) double‐positive newly generated neuroblasts, along with a pie chart showing the proportion.

We next explored the long‐term impact of adoptively transferred ChP ILC2 on brain function following TBI. scRNA‐seq analysis revealed that AREG expression in ChP ILC2 was notably higher in both abundance and proportion compared to other classical effector cytokines (IL5 and IL13) (Figure S6A, Supporting Information). Given the known reparative role of AREG in various tissue injury processes, we selected AREG as the key effector of ChP ILC2 for further investigation. To assess the contribution of AREG, we amplified and isolated ILC2 from AREG^−/−^ mice, yielding ILC2 that do not express AREG (ILC2^AREG‐KO^) (**Figure** **6**D). We transplanted both ILC2^WT^ and ILC2^AREG‐KO^ into the brain ventricles of mice 1 day prior to CCI modeling and performed histological analysis after evaluating the neurological function at 30 days post‐TBI (Figure 6D).

Compared with sensorimotor function, mice receiving ILC2 transplant showed significant recovery in long‐term spatial learning and memory, as evidenced by decreased latency to find the hidden platform and increased frequency of crossing the location of the removal platform in the Morris Water Maze (MWM) test (Figure 5A). The improvement in post‐traumatic spatial cognition was further verified by the “Y‐maze” test, where ILC2^WT^‐treated mice spent more time in and entered the novel arm more frequently (Figure 6B). In contrast, ILC2^AREG‐KO^ treatment failed to yield significant improvements in either the MWM or Y‐maze tests (Figure 5A,B). Furthermore, mice treated with anti‐CD90.2 mAb to eliminate ILC2 exhibited more severe spatial memory impairment in both MWM (Figure S7A–C, Supporting Information) and Y‐maze (Figure S7D,E, Supporting Information) tests compared to the IgG isotype treatment group.

To identify the structural basis for these differences in memory function, we performed histological staining of brain sections from sacrificed mice. Surprisingly, even though the cortical defect did not show improvement (Figure 6C,E), ILC2^WT^ transfer effectively preserved the integrity of the injured hippocampus, while ILC2^AREG‐KO^ did not (Figure 6C,F). Aside from the subventricular zone (SVZ), the DG of the hippocampus is the only region known to exhibit substantial neurogenesis in adults. The maintenance effect of ILC2 on hippocampal integrity likely benefits from both the optimization of the immune microenvironment and the acceleration of neurogenesis. Therefore, we used DCX to label neuroblasts, a key intermediate cell in hippocampal neurogenic progression.^[^ ^36^ ^]^ We observed a tremendous improvement in the quantity and arrangement of neuroblasts in the traumatic hemisphere (Figure 6C). However, the excessive hippocampal deletion in the vehicle and ILC2^AREG‐KO^ groups led to statistical issues (Figure 6C). Importantly, we also observed a significant expansion of DCX number (Figure 6C,G), synapse length, and branch number of neuroblasts in the hippocampus of the contralateral hemisphere following ILC2^WT^ treatment (Figure 6C,H,I). These findings suggest that the observed effects on neurogenesis were not solely due to injury‐induced processes but were further promoted by ILC2. Furthermore, ILC2^WT^ treatment increase the area of subgranular zone (SGZ) but did not alter the granule cell layer (GCL) volume (Figure 6J,K). Moreover, neuroblasts derived from the SVZ expanded significantly and migrated toward the injury site after ILC2^WT^ treatment (Figure S8A,B, Supporting Information), further highlighting the role of ILC2 in promoting neurogenesis across both neurogenic regions of the brain.

Interestingly, AREG deficiency in ILC2 reduced the proliferation of neuroblasts in both the DG and SVZ (Figure 6C,G–I), although it seemed to enhance the migration efficiency of neuroblasts from the SVZ to the injured region (Figure S8A,C, Supporting Information). To further elucidate the mechanism by which ILC2 promotes neurogenesis, we labeled cells with BrdU and Ki67. Both are markers of cell proliferation.^[^ ^37^ ^]^ The difference is that BrdU needs to be injected before testing, and it can mark newly generated cells; while Ki67 can be directly stained and detected, which indicates that the cells are dividing.^[^ ^37^ ^]^ The combination of the two markers can determine the progress of cell proliferation. The results showed that in the DG region of the hippocampus, BrdU labeling was almost entirely localized to neuroblasts (Figure 6L), while Ki67 showed little co‐localization with DCX (Figure S8D, Supporting Information). These results suggest that with the effect of ILC2, newly born neuroblasts (BrdU^+^) are indeed generated, which may not benefit from the proliferation of neuroblasts themselves, but the cell expansion in the neurogenetic lineage more upstream. The definition of lineage cells and pseudotime analysis in the context of single‐cell data are effective means to reveal this phenomenon.

### 2.9 ILC2 Colonized in ChP Motivate the Initiation Steps of Neurogenesis in the DG of the Hippocampus at the Early Phase After TBI in an AREG/EGFR Pathway Dependent Manner

**Figure 7:**
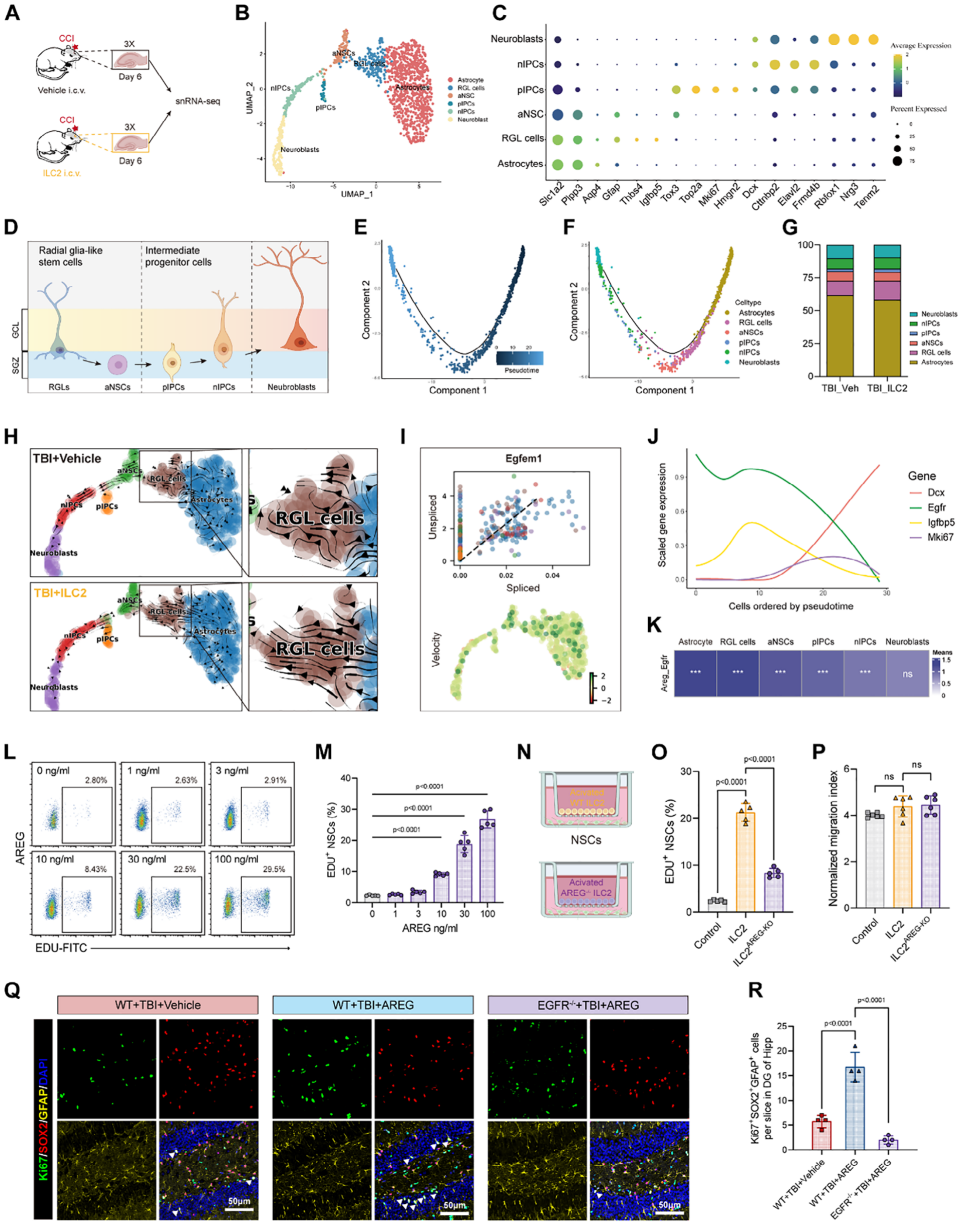
ILC2 colonized in ChP motivate the initiation steps of neurogenesis in the DG of the hippocampus at early stage after TBI in an AREG/EGFR pathway dependent manner. A) Experimental design for the snRNA‐seq of hippocampus. B) UMAP plots of 6 sub‐clusters for neurogenesis from vehicle and ILC2 treated groups. C) Dot plots showing the average expression (color) and the percentage of expressing cells (circle size) for characteristic marker genes (columns) in each sub‐cluster. D) Schematic diagram of neurogenesis in the DG of the hippocampus. E) Cells from all NSCs sub‐clusters along the pseudotime trajectory. F) Map for pseudotime trajectory results of NSCs. G) Proportion of 6 sub‐clusters involved in pseudotime analysis from vehicle and ILC2 treated group after TBI. H) RNA velocity analysis mapped in UMAP space for 6 sub‐clusters involved in neurogenesis. The arrows in the figure are merged vectors of multiple single cell vectors with similar locations and future differentiation states. I) One of the top 6 key genes mediating RNA velocity in RGLs cluster with the treatment of ILC2 after TBI, as well as corresponding RNA velocity UMAP. For the rest of 5 key genes see also Figure S7E. J) Relative expression of specific genes across the cells of the neurogenic lineage arranged by pseudotime. K) The heatmap shows the strength of AREG‐EGFR interaction in ILC2 and 6 clusters of the neurogenic lineages based on CellPhoneDB analysis. L,M) Representative flow cytometry pseudocolor images and proportions of newly proliferated NSCs (EDU^+^) with the treatment of different concentration of AREG. n = 5. N–P) Schematic diagram of NSCs and ILC2 co‐culture system (N), and the proliferation proportion (O) and migration distance (P) of NSCs with activated ILC2 stimulation. n = 6. Q,R) Quantitative comparison of the numbers of GFAP^+^SOX2^+^Ki67^+^ early stage cells of neurogenic lineage in the injured DG of the hippocampus from WT or EGFR^−/−^ mice. n = 4.

To explore the alterations in the neurogenic lineage induced by ILC2 treatment, we performed single‐nuclear RNA sequencing (snRNA‐seq) of the hippocampus from the injured hemisphere at 6 days post‐TBI (**Figure** **7**A). Compared to scRNA‐seq, snRNA‐seq offers greater specificity for cells of the neural lineage.^[^ ^38^ ^]^ Neurogenesis in the DG of the hippocampus is known to follow a stepwise differentiation process from radial glial‐like (RGL) neural stem cells, through intermediate progenitor cells, neuroblasts, and ultimately mature neurons^[^ ^39^ ^]^ (Figure 7D). We distilled neurogenic lineage cells in all major clusters from snRNA‐seq results according to the relative spatial relationship (Figure S9A, Supporting Information) and specific characteristic genes related to neural proliferation and regeneration (e.g., *Sox11*, *Dcx*, *Stmn1*, and *Ccnd2*)^[^ ^40^ ^]^ (Figure S9B, Supporting Information). As a result, major cluster Astrocyte/RGL cells and neural precursor cells were selected for further re‐clustering, which revealed 6 sub‐clusters: astrocytes, RGL cells, activated neural stem cells (aNSCs), proliferating intermediate progenitor cells (pIPCs), neuronal intermediate progenitor cells (nIPCs) and neuroblasts (Figure 7B).

As reported previously, it is difficult to differentiate RGL cells from astrocytes, as they share similar expression profiles^[^ ^40^, ^41^ ^]^ (e.g., *Gfap*, *Slc1a2*). Here, we also fitted astrocytes into the neurogenic lineage and distinguished these two sub‐clusters with specific markers. *Aqp4*, a marker of activated astrocytes, is indicated to be barely detected in RGL cells (Figure 7C). Additionally, we defined two genes (*Thbs4* and *Igfbp5*) with stem cell specificity as markers for RGL cells combining previous literature^[^ ^41^, ^42^ ^]^ (Figure 7C). Adjacent to RGL cells, we appoint *Tox3* to identify aNSCs and IPCs that had entered the active differentiation program, while pIPCs displayed significant proliferation markers such as *Mki67* and *Hmgn2* (Figure 7C). As differentiation proceeded, nIPCs and neuroblasts expressed genes associated with neuronal commitment, particularly *Dcx* (Figure 7C). The changes in marker gene expression across these six sub‐clusters were visualized by VlnPlot (Figure S9C, Supporting Information).

Additionally, we performed pseudotime analysis of all neurogenetic lineage cells and astrocytes to reveal the differentiation trajectory during the subacute phase after TBI (Figure 7E). By labeling neurogenetic lineage cells with real clustering information, we found that the sub‐clusters corresponded well with the pseudotime analysis, confirming their expected differentiation trajectories (Figure 7F). To assess the impact of ILC2 treatment, we compared the proportions of each sub‐cluster between vehicle and ILC2‐treated groups. While there were no significant changes in the proportions of most sub‐clusters, RGL cells showed a modest, but non‐significant, increase in the ILC2‐treated group (Figure 7G).

We subsequently implemented RNA‐velocity analysis to estimate the future state of each cell by comparing spliced and unspliced transcripts. This analysis provided further evidence of the differentiation trajectory and highlighted the enhanced proliferative capacity of RGL cells following ILC2 treatment, as indicated by the increased arrow vectors in the same direction within RGL cells (Figure 7H). Gene Ontology (GO) enrichment analysis of ILC2‐treated RGL cells revealed enhanced functions related to brain development, neuroblast division, and DNA repair (Figure S9D, Supporting Information). These findings suggest that ILC2 treatment significantly promotes the proliferation potential of RGL cells at 6 days post‐TBI, although the expansion of these cells and their downstream progeny has not yet occurred at this early time point.

To further investigate whether AREG signaling mediated the effects of ILC2 on RGL cells, we identified the top six genes that regulate the RNA‐velocity of RGL cells, in order of importance:  *Elavl2*, *Egfem1*, *Akap9*, *Prox1*, *Dpp6*, and *Map2* (Figure 6I; Figure S9E, Supporting Information). EGF‐like and EMI domain containing 1 (*Egfem1*) is a pseudogene, which is unable to encode a functional protein. However, combined with the high similarity and gene proximity between pseudogenes and normal genes, we speculate that EGF family factors may play a critical role in the RNA‐velocity regulation of RGL cells treated with ILC2. As a member of the EGF family, AREG can bind to EGFR to elicit its effects. Interestingly, EGFR is highly expressed in early stages of the neurogenic lineage cells, particularly RGL cells, which is manifested by both expression of feature gene in pseudotime and VlnPlot arranged with cell types (Figure 7J; Figure S9C, Supporting Information). In addition, the combined analysis of ChP scRNA‐seq data and hippocampal snRNA‐seq data showed a significant interaction between ILC2 derived AREG and EGFR on RGL cells (Figure 7K).

We next validated the role of AREG in promoting NSC proliferation in vitro. Recombinant AREG (rAREG) was added to NSCs isolated from neonatal mice, and cell proliferation was evaluated by EDU incorporation. The results showed a significant, concentration‐dependent increase in NSCs proliferation with AREG treatment (Figure 6L,M). Second, we co‐cultured NSCs with WT ILC2 and AREG^−/−^ ILC2 respectively, and found that WT ILC2 also remarkably promoted the proliferation of NSCs in vitro, while no such effect was observed with AREG^−/−^ ILC2 (Figure 7N,O). Furthermore, co‐culturing with ILC2 did not seem to alter the migration ability of NSCs in vitro (Figure 7P).

Finally, we examined the effects of i.c.v. injection of rAREG in WT and EGFR^−/−^ mice. Early neurogenic lineage cells represented as RGL cells were successfully identified according to Gfap and Sox2 double positive and bipolar morphological characteristics (Figure 7Q). We further labeled the proliferation characteristics of these cells with Ki67, and the results demonstrated that on the 6th day after TBI, the proliferating RGL cells (GFAP^+^Sox2^+^Ki67^+^) in the hippocampus DG region of the mice treated with AREG were significantly abundant than those in the vehicle‐treated group. As expected, AREG failed to promote RGL cell proliferation in EGFR^−/−^ mice.

## 3 Discussion

This study elucidates a neuroprotective mechanism in which ILC2, anchored and expanded within the ChP fibroblast niche, generates an AREG‐enriched CSF that reduces acute inflammatory infiltration, maintains hippocampal integrity, and improves neurological deficits during both acute and chronic phases following TBI. First, we confirmed that fibroblasts are the principal source of IL‐33 in the ChP, and the upregulation of IL‐33 contributes significantly to the expansion of ILC2 in the ChP post‐TBI. The spatial structure of ILC2 within the ChP fibroblast niche was characterized, and we identified the VCAM‐1/Integrin α4β7 pathway as a key mediator of this anchoring effect. Second, our data demonstrated that induced expansion or adoptive transfer of ILC2 in the ChP suppresses immune infiltration and promotes neurogenesis during the acute phase of TBI, thus playing a critical role in maintaining hippocampal integrity and improving memory impairment in the chronic phase. Finally, we uncovered the potential mechanism by which ILC2 mediate neurogenesis in the DG of the hippocampus during the chronic phase of TBI: ILC2‐secreted AREG interacts with RGL cells to promote their mitosis, thereby initiating differentiation during the early phase after TBI.

ChP is a tiny and deeply located CNS‐associated border immune site, which has garnered increasing attention in recent years.^[^ ^43^, ^44^ ^]^ Under normal conditions, the primary immune cells in the ChP are resident macrophages (also referred to as border‐associated macrophages), followed by T cells, with other immune cell types present in rare proportions.^[^ ^3^, ^43^ ^]^ Recent studies have revealed crosstalk between the ChP epithelium and resident macrophages during acute injury, such as intraventricular hemorrhage, leading to a cascade of inflammation and high secretion of epithelium.^[^ ^43^, ^45^ ^]^ The ChP has also been shown to be a critical site for T‐cell infiltration into the brain during conditions such as multiple sclerosis (MS) and other infectious diseases.^[^ ^28^, ^46^ ^]^ However, the immune landscape of the ChP after brain injury, particularly in the chronic phase, has not been systematically explored. We reveal the cellular dynamics of the ChP following TBI through mixed scRNA‐seq, identifying a largely uncharacterized, specialized population: ILC2. Although the ILC2 population is small, our results build upon Ivan et al.’s work, which previously observed ILC2 exclusively in the ChP of aging mice.^[^ ^24^ ^]^ Notably, we found that the abundance of ILC2 gradually increases over time in the ChP following TBI, becoming the sole immune cell population upregulated during the chronic phase, suggesting its pivotal role in the protracted neural repair process. Similar immune regulatory signals emanating from the ChP have been reported in relation to neural repair and development. For instance, Violeta et al. pointed out that a series of factors from the aging ChP regulate NSCs in the SVZ region through CSF as a mediator.^[^ ^6^ ^]^ The Michal Schwartz team has demonstrated in two independent studies that M2 macrophages recruited by the ChP, or IFN‐γ signaling from the ChP, promote spinal cord repair.^[^ ^7^, ^28^ ^]^

The existence of ILC2 in normal mouse brain tissue remains a topic of debate.^[^ ^30^ ^]^ The prevailing view, supported by our data, suggests that ILC2 are absent from the brain parenchyma of healthy mice.^[^ ^21^ ^]^ This raises significant questions about the pathways and locations through which ILC2 infiltrates the brain under pathological conditions. Both TBI and hemorrhagic stroke are associated with the potential for direct immune cell infiltration due to vascular rupture. Notably, the study by Pei Zheng et al. provides partial evidence for the accumulation of ILC2 in the brain parenchyma of middle cerebral artery occlusion (MCAO) mice.^[^ ^22^ ^]^ This finding, in a non‐hemorrhagic injury model, implies the existence of alternative intracranial pathways for ILC2 infiltration. For the first time, we establish a direct link between ChP fibroblasts and ILC2, evidenced by a specific “niche” structure formed by fibroblasts to support ILC2 expansion. This interaction is mediated by the IL‐33/ST2 pathway for ILC2 expansion and the VCAM‐1/Integrin α4β7 pathway for anchoring ILC2 in the ChP stroma. Fibroblast‐like cells, which are important sources of IL‐33 and TSLP, have been shown to provide essential sites for the development and activation of ILC2 in various tissues, such as the thymus, blood vessel adventitia, skeletal muscle, and lung.^[^ ^11^ ^47^, ^48^ ^]^ The role of activated ILC2, however, varies by tissue type and disease context. For example, adventitial fibroblast‐ILC2 niches have been reported to suppress liver fibrosis,^[^ ^49^ ^]^ while in Duchenne muscular dystrophy, ILC2 are involved in fibrotic processes in skeletal muscle in an eosinophil‐dependent manner.^[^ ^11^ ^]^ In the context of TBI, our results demonstrate that ILC2 significantly attenuate immune cell infiltration during the acute phase, a pattern similar to the immunosuppressive effects of ILC2 in acute liver and kidney injury, as well as in hemorrhagic and ischemic strokes.^[^ ^15^, ^16^, ^22^, ^23^ ^]^ Importantly, we emphasize that ILC2 do not reside in the brain parenchyma during acute injury unless externally administered, as confirmed by careful exclusion of ChP contamination during sample preparation.

We further show that ChP‐resident ILC2 significantly improve memory performance 30 days after TBI, which is attributed to the maintenance of hippocampal integrity. Interestingly, we found that ILC2 did not improve the repair of the cerebral cortex. This aligns with Ivan et al.’s observation that ILC2 in the aging ChP can alleviate age‐related memory decline.^[^ ^24^ ^]^ Our study provides novel evidence that the biological basis of ILC2‐mediated memory improvement is linked to the promotion of neurogenesis. Previous studies have suggested that regulatory T cells (Tregs), which share functional similarities with ILC2, regulate NSCs proliferation in the SVZ following ischemic stroke.^[^ ^50^ ^]^ More recent work has shown that meninges‐resident Tregs support adult hippocampal neurogenesis. It is theoretically highly possible that ILC2 residing in the ChP regulates hippocampal neurogenesis,^[^ ^39^ ^]^ as the spatially closer proximity between the ChP and the DG region of the hippocampus. Thus, the therapeutic effect of ILC2 in the hippocampus may be attributed to a combination of improved immune microenvironment and enhanced neurogenesis.

The targeted elimination of ILC2 plays a pivotal role in this study. We successfully eliminated ILC2 in the ChP following TBI through i.p. injections of the anti‐CD90.2 mAb every other day. This intervention led to an exacerbation of the inflammatory storm post‐TBI, accompanied by worsened motor‐sensory function and spatial memory impairment. Similar findings have been reported in prior research on hemorrhagic stroke.^[^ ^23^ ^]^ However, a study suggests that treatment with anti‐CD90.2 mAb does not effectively eliminate meningeal ILC2.^[^ ^21^ ^]^ We hypothesize that anti‐CD90.2 mAb may not affect physiologically colonized ILC2 but instead inhibits the reactive proliferation and migration of ILC2 in lymphoid organs. Notably, CD90.2 is also expressed in CD4^+^ helper T cells, mesenchymal stromal cells, neural cells (e.g., retinal ganglion cells), and other innate lymphocyte subtypes, such as ILC1 and ILC3.^[^ ^51^ ^]^ According to prior studies, treatment with anti‐CD90.2 mAb was observed to reduce the number of helper T cells,^[^ ^14^, ^52^ ^]^ potentially explaining the observed reduction in CD4^+^ T cells within injured hemisphere. Regarding ILC1 and ILC3, we mainly excluded its potential role based on our single‐cell analysis of ChP tissue from sham and TBI, where we did not classify such cells. Collectively, these findings suggest that the exacerbation post‐TBI associated with anti‐CD90.2 mAb administration is, at least in part, attributable to the depletion of ILC2.

At present, the definition and classification of neurogenic lineage cell types remain controversial. RGL cells are often considered synonymous with NSCs in certain definitions.^[^ ^53^, ^54^ ^]^ AREG has been recognized as an effective mitogen for NSCs and shows broad tissue repair effects.^[^ ^18^, ^55^ ^]^ AREG not only binds EGFR but also upregulates VEGF production and the release of TGF‐β, both of which are involved in tissue repair.^[^ ^56^ ^]^ ILC2‐derived AREG also showed superior repair function in lupus nephritis, atretic biliary epithelium, and so on.^[^ ^31^, ^57^ ^]^ In this study, we defined RGL cells through snRNA‐seq and immunofluorescence staining and demonstrated that AREG secreted by ILC2 induces RGL cell proliferation, a critical step in promoting neurogenesis.

There are several limitations to our study. First, we encountered challenges in constructing ILC2‐specific knockout transgenic animals to directly demonstrate the unique role of ILC2 in neuroinflammation and neurogenesis. Although we utilized anti‐CD90.2 mAb to deplete ILC2, this approach inadvertently led to the depletion of T cells as well, making it difficult to conclusively determine whether ILC2 are essential for neural protection following TBI. Second, although the expression levels of Il5 and IL13 in ChP ILC2 after injury were significantly lower than that of AREG, further comprehensive validation is needed to rule out the possibility that these two molecules may also serve as effector molecules mediating ILC2‐driven neuroprotection. Third, our study did not thoroughly investigate the potential role of the 4 V ChP in inflammation suppression and neurogenesis promotion. As demonstrated by Neil Dani et al., the 4 V ChP plays a significant role in hindbrain development, and its volume is notably larger than that of the other two ChP. This suggests that although spatially distant from the hippocampus, the 4 V ChP may still possess substantial regulatory potential over hippocampal function. Future studies may require more detailed separate investigations of each ChP region. Regardless, the salient finding is that ChP ILC2 safeguards hippocampal integrity and memory in mice that have undergone TBI in a multipronged fashion.

In conclusion, our findings show that ILC2 significantly expands within the ChP fibroblast niche after TBI, inhibiting excessive immune infiltration and promoting neurogenesis, ultimately maintaining hippocampal integrity and alleviating memory deficits. AREG has been identified as a key mediator between ILC2 and RGL cells, initiating the differentiation of neurogenetic lineages. These results provide novel insights into the regulatory role of the ChP in neuroprotection and tissue repair following brain injury.

## 4 Experimental Section

### Human CSF Sample Collection

CSF from the PTH group was collected from patients exhibiting ventricular system expansion on CT imaging within two weeks following severe TBI. CSF from the NPH group was obtained from patients with unexplained intracranial hypertension, after excluding predisposing factors such as trauma, tumor, subarachnoid hemorrhage, CNS infection, and others. CSF was collected incidentally during diagnostic or therapeutic lumbar punctures or ventricular shunt procedures. All samples were sealed and stored at −80 °C for subsequent analysis.

### ELISA Assay

The concentrations of IL‐33 in human CSF were measured using an ELISA kit (D3300B, R&D Systems, USA) following the manufacturer's instructions.

### Experimental Animal Studies

C57BL/6J, Il33^−/−^, Areg^−/−^, Egfr^−/‐^ mice were purchased from the GemPharmatech Co. Jiangsu. After adapting to the new environment for 1–2 weeks, male mice (8–10 weeks old) were used for in vivo experiments. All animals were accommodated in a facility with controlled temperature and humidity and a 12‐h light/dark cycle. Food and water were provided ad libitum. All mice were randomly assigned to receive CCI modeling or subsequent treatment.

### TBI Modeling

The TBI Model of mice was constructed with the CCI method as previously reported.^[^ ^58^ ^]^ Roughly, after anesthetized with sodium pentobarbital, mice were fixed on a stereotactic skull frame. After fully exposure, a bone window was craniotomized on the right parietal bone with a grinding drill (centered 1 mm anterior and 1.5 mm lateral to bregma; diameter: 4 mm). The Benchmark electromagnetic cranial trauma impactor was used to create a CCI mouse model by impacting the cerebral cortex with an application of force (velocity: 3 m s^−1^, depth: 1.5 mm, duration: 120 ms). In the sham group, a craniotomy was performed without applying impact. All mice were monitored closely until fully recovered from anesthesia.

### Intracerebroventricular (i.c.v.) Injection and i.c.v. Trocar System

Total dose of 15 µg/5 µL rAREG (989‐AR, R&D Systems) or vehicle was i.c.v. injected as follows: after being anesthetized and fixed, a hole was drilled for the insertion of a fine needle (0.4 mm posterior to the bregma; 1 mm lateral to the sagittal suture; depth: 2.5 mm; speed: 1 µL/1 min). For the ***AAV2/5 infection***, 5ul of AAV‐IL33 (pcAAV‐CMV‐Il33‐3xFLAG‐P2A‐GdGreen‐WPRE, OBiO Technology, CHN) and AAV‐control (pcAAV‐CMV‐GdGreen‐WPRE, GL3048, OBiO Technology, CHN) with titer of 2.48E+13 were i.c.v. injected in the method described above respectively. In the end of the injection, it is necessary for the needle to stay for an additional 5–10 min. The AAV injected into mice used for FACS and immunofluorescence detection is a non GdGreen‐labeled AAV2/5, so as to avoid false‐positive signals during detection. For ***i.c.v. trocar system***, the basic steps and positioning are consistent with i.c.v. injection. The difference is that a special ***trocar*** is inserted into the ventricle, whose tail end is fixed on the skull surface by a base with glue to prevent displacement. The external core needle for injection was repeatedly punctured into the ventricle through this the ***trocar*** to achieve multiple injections of rIL33 for consecutive days. The injection system can be firmly connected to the base to facilitate the free movement of mice and reduce the impact of the operation.

### Behavioral Tests

A series of behavioral tests were used to assess the subacute sensorimotor deficits and chronic memory deficits after TBI as described previously.^[^ ^59^, ^60^ ^]^

### Body Curl Test

The body curl test was performed to assess contralateral torso flexion. In brief, mice were hand‐suspended by the tail, and their trunk bending degree was scored according to the following criteria: 1) deviating 0° from vertical (no flexion); 2) Torso bending 22.5 degrees or less; 3) flexion from 22.5° to 45° from vertical; 4) flexion≥ 45°and with or without grasping of the hindlimbs by forelimbs. Three independent trials per day were completed as soon as possible when the mice were picked up for other behavioral tests.

### Foot Fault Test

To evaluate sensorimotor coordination, mice were placed on an elevated grid surface with a grid opening of 2.5 cm^2^, with a video below recording for 2.5 min. All recorded videos were analyzed by a blinded researcher to mark and calculate the percentage of foot fault in total. Foot faults are defined as impaired side limbs (left forepaw or left hind paw; contralateral to CCI hemisphere) that fell through the grid.

### Cylinder Test

To evaluate forelimb asymmetry, the free movement of mice was recorded in a transparent cylinder (diameter 10 cm; height 17.5 cm) with a video camera for 15 min. When probing the cylinder, mice normally show spontaneous rears touching the cylinder wall with the left forelimb (L), the right forelimb (R), or both forelimbs (B). Another blinded investigator analyzed the videotapes to asymmetry rate, which manifested as (R‐L)/(L+R+B) x100%.

### Adhesive Removal Test

To assess sensitivity and motor deficit of forepaw, a piece of adhesive tape (0.1 cm^2^) was adhered to the left forepaw (injured side; contralateral to CCI hemisphere) of mice and recorded the latency to completely remove it, with a maximum of 1 min. Mice were trained for 3 consecutive days before the CCI modeling. Trials were repeated 3 times on each testing day and recorded as the average latencies.

### Rotarod Test

Motor coordination and balance deficits were assessed on an accelerating Rotarod (RWD Life Science). Mice were placed on a rotating drum for 5 min with the speed accelerating from 4 to 40 rpm. Mice were trained for 3 consecutive days before the CCI modeling. For each day mice were tested 3 times (more than 10‐min interval). Each latency to fell off during the training and testing period was recorded and averaged for statistical analysis. This test is the last item on each experimental day, as it takes a long time and will force the mice to a short state of exhaustion, which may affect the results of other tests.

### Morris Water Maze Test

Cognitive deficits were assessed with the Morris water maze test, as described previously.^[^ ^58^ ^]^ Briefly, a round platform (diameter: 11 cm) was submerged 1.5 cm beneath the water surface in a circular pool (diameter: 109 cm). The pool was filled with water (temperature: 20 ± 1 °C) mixed with white MgO_2_. Mice were put into the pool along the wall from the third quadrant (farthest from the first quadrant where the platform is located) and allowed to search for the hidden platform for 1 min. Mice were pretrained for 5 consecutive days (25–29 days after TBI) before the final test. The swimming trajectories of mice were recorded by the animal behavior analysis system (Zhenghua, Anhui) and the latency to find a platform was derived to reflect spatial learning (the average of three independent experiments). Mice were allowed to stay on the platform for 15 s to deepen the memory of the platform location. For the final test (30 days after TBI), the platform was removed, and the mice were put into the pool in the same position as the training phase. The trajectory of the mice was recorded with the analysis system, which produced the time staying in the first quadrant, the times of crossing the potential platform, and the speed of swimming reflected spatial memory.

### Novel Y maze

Cognitive deficits was additionally assessed with the novel Y maze, as modified recently.^[^ ^39^ ^]^ In brief, three arms equally divided at an angle of 120° were placed in a space without external interference, in which the gate of the “novel” arm was blocked to prevent mice from entering. When pre‐training began, the mice were placed into the “start” arm, and were allowed for free movement in the “start” arm and the “familiar” arm for 7 min to familiarize itself with the environment. In the testing phase, the gate of the “novel” arm was removed, and healthy mice would have a stronger desire to explore the unfamiliar “novel” arm thus staying in it for a longer time. The analysis system was used to record the trajectories of the mice and derive their dwell time in the “novel” arm and the frequency of entries into the novel.

### ChP Tissue Dissection and Dissociation

After deep anesthesia, the mice underwent whole‐body perfusion with ice‐cold phosphate‐buffered saline (PBS) before brain tissue harvesting. All ChP tissues were dissected from the corresponding ventricles (LV, 3 V and 4 V) with a microscope, according to the previously reported anatomy and procedures. The ChP tissue were further microdissected in ice‐cold DMEM and digested at 37 °C for 30 min (invert and shake every 5 min) with Liberase TM (5 ug mL^−1^, 5401119001, Roche, GRE) and DNaseI (45 U mL^−1^, 10104159001, Roche, GRE). The entire enzymatic system was terminated with twice the volume of ice‐cold PBS, filtered through a 70‐µm cell strainer (Biosharp, CHN), followed by centrifugation at 340 g, 4 °C for 5 min. Resuspend the bottom cells in FACS buffer for subsequent single‐cell sequencing and flow cytometry.

### Flow Cytometry Analysis

Flow cytometry analysis was performed with ChP samples and brain tissue from injured hemisphere samples. Single‐cell suspensions of ChP were prepared as described above. single‐cell suspensions of brain tissue were prepared with mechanical dissociation method, as described previously. In brief, after sufficient perfusion with ice‐cold PBS, the left hemisphere (injured site) was harvested and finely ground in a homogenizer to get the homogenate. the homogenate was filtered using a 70‐µm cell strainer, then mixed with 30% Percoll (17089109, Cytiva, USA), and then the myelin sheaths were removed using density gradient centrifugation. After lysing red blood cells with 1×RBC LYSIS BUFFER SOLN (00‐4333‐57, Thermo Fisher, USA), suspensions were incubated with CD16/32 antibody (Biolegend, USA) for Fc receptors blocking. Cells were first incubated with Zombie NIR (423106, BioLegend, USA) for 15 mins to evaluate the viability, and then surface antibodies (Table S1, Supporting Information) were incubated in the dark for another 30 mins at 4 °C. For detection of the intracellular IL5, IL10, and AREG levels, cells were fixed and permeabilized with an Intracellular Fixation and Permeabilization Buffer Set (88‐8824‐00, Thermo Fisher, USA), then incubated for 30 mins with corresponding antibodies. Flow cytometry was performed on the CytoFLEX flow cytometer (Beckman), and the data were analyzed using the FlowJo software.

### ILC2 Isolation, Expansion and Adoptive Transfer

ILC2 were isolated by Fluorescence Activated Cell Sorting (FACS). Briefly, spleen, mesenteric lymph nodes (MLNs) and bone marrow were harvested from uninjured mice treated with IL33 (400 ng per day, i.p.) daily for 7 consecutive days. After grinding and filtering through a 70‐µm cell strainer, the density gradient centrifugation was mediated by Ficol (Cytiva, USA) and red blood cell lysis. All prepared single‐cell suspensions were pooled together. Cell suspensions were first subjected to Magnetic activated Cell Separation (MACS) using a lineage cell depletion kit (Miltenyi Biotec) to remove lineage cells and improve the efficiency of FACS. With the incubation of flow cytometry antibody, CD45+Lineage‐CD127+ST2+ ILC2 were isolated using CytoFLEX SRT flow cell sorter (Beckman). The isolated ILC2 must be transferred as soon as possible or expanded in serum‐containing 1640 medium supplemented with activating factor including IL‐2 (10 ng mL^−1^, Biolegend), IL‐7 (10 ng mL^−1^, Biolegend), and IL‐33 (50 ng mL^−1^, Biolegend) for at least 1week. For in vivo studies, 2 × 105 ILC2 in 5 µL media were i.c.v. transferred to recipient mice 1 day before CCI modeling.

### ILC2 Depletion

In order to deplete the ILC2, anti‐CD90.2 mAb (105302, BioLegend, USA) was i.p. injected into mice at a dose of 300 µg every two days until sacrificed as previously described.^[^ ^14^, ^22^, ^23^ ^]^

### Immunohistochemistry and Image Analysis

Mice were transcardially perfused with ice‐cold PBS followed by 4% PFA. Brains were harvested, kept in 4% PFA 4 °C overnight and gradient dehydrated with 15% and 30% sucrose solution. The samples were frozen in Optimal Cutting Temperature (OCT) compound and carefully sliced into 12‐µm sections to ensure the integrity of the ChP. For the whole ChP dyeing preparation, ChP were microdissociated from brain after 4%PFA fixation for one night. The slices or ChP tissue were blocked and permeabilized with QuickBlock Blocking Buffer (P0260, Beyotime, CHN) for 45 mins. The samples were incubated with primary antibodies (Table S1, Supporting Information) at 4 °C overnight in the chromatography cabinet and then with species corresponding fluorescent secondary antibodies (Thermo Fisher, USA). 0.01 M PBS was used for thorough washing between each staining. The samples were mounted with DAPI‐containing anti‐fluorescence quenching agent (ab104139, Abcam, UK) and photographed by Lecia DMI8 confocal microscope. For GATA3 and Ki67 staining, sections must be boiled in a sodium citrate solution for 12 min for antigen retrieval. For BrdU staining, the section must be acidified with 0.01 M HCL at 37 °C for 30 min to expose its antigenic sites.

For the quantitative analysis of CD68 and Iba‐1 double‐positive microglia in the hippocampal DG region, consistent ROI selection criteria were maintained: specifically targeting the junctional area between the GCL and SGZ, while ensuring these structures remained centered in all imaging fields.

For image analysis, slices used for analysis are selected from the corresponding layers based on the morphology of LV and 3 V and the morphology of the hippocampus. Two independent observers blinded to grouping analyzed the area of the defect, GCL volume, SGZ area, DCX^+^ cell number, and the length and number of branches of neuroblasts in Image J software.

### BrdU Injections

To label newborn cells, mice were i.p. injected with the thymidine analog 50‐bromo‐20‐deoxy‐uridine (BrdU, 50 mg kg^−1^, B5002, Sigma, GRE) for 3 consecutive days, beginning at 3 days after CCI modeling.

### Real‐time PCR and Western blot

These two methods were not the main research technique in this study, so only some special items are stated. All ChP tissues in LV, 3 V and 4 V of each mouse were harvested for protein or RNA extraction as one sample. For real‐time PCR anaylysis, RNeasy Mini Kit (QIAGEN) was used for the extraction of trace RNA from ChP according to the manufacturer's instructions, and pre‐designed Taqman (ThermoFisher) primers including Il25 (Mm00499822_m1), Il33 (Mm00505403_m1), Tslp (Mm01157588_m1) were used. For Western blot analysis, the tissues were placed in 50 µL of cell lysis buffer (P0013, Beyotime) and sonicate for 25 s, and the antibody used was Goat anti‐IL33 antibody (AF3626, R&D Systems).

### Primary Murine NSCs Culture

Primary NSCs were obtained from adult (5–6 weeks) mice and neurosphere cultures were conducted as reported previously.^[^ ^61^ ^]^ The tissues of the SVZ region and the DG region of the hippocampus were disserted from the harvested whole brain. After mechanical dissociation, the fragments of tissue were digested at 37 °C for 5 min with StemPro Accutase (07922, STEMCELL Tech.). Followed by filtering with a 40‐µm strainer, the purified NSCs were inoculated at a density of 2 × 10^5^ cells mL^−1^ in NeuraCult proliferation medium (05702, STEMCELL Tech.) supplemented with EGF (20 ng mL^−1^, 351‐09, PeproTech), bFGF (20 ng mL^−1^, 450‐33, PeproTech), and necessary heparin solution (0.1%, 07980, STEMCELL Tech.) to prevent NSCs agglutination. NSCs were passaged every 3 days, and p2‐4 NSCs were depleted of EFG and treated with different concentrations of rAREG (989‐AR, R&D Systems, USA) or cocultured with isolated ILC2 in a transwell insert.

### EdU Proliferation Assay

The proliferation of NSCs was assessed with an EdU Staining Proliferation Kit (ab219801, Abcam, UK) as the previous work described.^[^ ^61^ ^]^ NSCs that undergone the ILC2 or rAREG treatment were incubated with 10 µm EdU for 3 h. Followed by digestion fixation, and permeabilization, NSCs were incubated with 488‐iFluor for 30 mins to label EdU. Using flow cytometer analysis, NSCs proliferating phase were characterized by EdU‐positive.

### NSCs Migration Assay

The migration of NSCs was conducted as described preciously. In brief, neurospheres were seeded onto 48‐well plates coated with 100 µg mL^−1^ polyD‐lysine (PDL, P6407, Sigma, GRE) and 10ug mL^−1^ of Aggrecan (A1960, sigma, GRE) in medium containing growth factors as described above. Followed by coculture with ILC2^WT^ or ILC2^AREG‐KO^ at 37 °C for 21 h, each well was photographed by DMi8 widefield microscope. The ratio of the total area of migrated cells and the inner area of neurospheres was defined as the Migration Index.

### Single‐cell RNA Sequencing

ChP cell suspensions were prepared as described above. For scRNA‐seq, 10 slices of ChP from the injured hemisphere were merged as one sample. Fresh suspensions were added to the 10x Chromium chip according to the instructions for the 10X Genomics Chromium Single‐Cell 3′ kit (V3). Libraries were sequenced by LC‐Bio Technology (Hangzhou, China) on an Illumina NovaSeq 6000 sequencing system (double‐end sequencing, 150 bp) with more than 20 000 reads depth per cell.

### Single‐nucleus RNA Sequencing

Fresh homogenate of hippocampus tissue (3 injured hippocampus for one sample) was prepared using a homogenizer (Dounce, Sigma) and debris was removed using Debris Removal Solution kit (Miltenyi). Nuclei were isolated with Nuclei EZ Lysis buffer (NUC‐101; Sigma‐Aldrich) supplemented with protease inhibitor (5892791001, Roche, GRE) and DNaseI (10104159001, Roche, GRE). The subsequent processes of cell capture and sequence are consistent with single‐cell RNA sequencing.

### Statistical Analysis

Results are presented as mean ± standard deviation (SD). Shapiro‒Wilk and Levene methods were used respectively to examine the normality and the homogeneity of variance of all data. Student's *t*‐test (2 groups) or one‐way analysis of variance (ANOVA) (≥ 3 group) were used for comparison of continuous variables with normality, followed by Tukey's post hoc test. Mann–Whitney test (2 groups) and Kruskal‒Wallis test (≥ 3 group) were used for comparison of variables without normal distributions, followed by Dunn's post hoc test. Data two independent variables were analyzed by two‐way ANOVA followed by the Sidak post hoc test. Generally, P values less than 0.05 are considered to be statistically significant, and all the P values in the corresponding statistical graphs were marked as much as possible. All statistical analyses were conducted with GraphPad Prism software (version 8.3.0).

### Single‐Cell/Single‐nucleus RNA Sequencing Data Analysis

The results obtained from Illumina sequencing offline were transformed into FASTQ format by means of bcl2fastq software (version 5.0.1). The sc/snRNA‐seq sequencing data were compared against the reference genome with the use of CellRanger software, and the 3′ end transcripts of cells and individual cells were identified and counted in the sequenced samples. The expression profile matrix output by CellRanger was imported into Seurat (version 4.1.0) for the filtration of low‐quality cells from scRNA‐seq data. Subsequently, the filtered data was downscaled and clustered. The thresholds for filtering low cell quality were as follows: the number of genes expressed per cell exceeded 500, and mitochondrial genes were expressed in less than 25% of cells. Cells were projected into a 2D space through t‐SNE or UMAP. These steps included: 1. Calculating gene expression values by means of the LogNormalize method within Seurat's “Normalization” function; 2. Performing principal component analysis (PCA) using the normalized expression values, and employing the top 20 PCs for clustering and Findcluster analysis. 3. Analyzing the marker genes of each cluster based on Findallmarker, and the marker genes were selected in accordance with the following criteria: expressed in over 10% of cells in each cluster, with a P value less than or equal to 0.01, and a gene expression ploidy logFC greater than or equal to 0.26.

### Cell–Cell Communication Analysis

Communication analysis between ILC2 and fibroblasts were performed with CellPhoneDB analysis and verified by calculating the ligand‒receptor score with SingleCellSignalR (v1.4.0). Interaction between ChP ILC2 and cells of neurogenetic lineage were analyzed by CellPhoneDB analysis only.

### RNA Velocity Analysis

To calculate RNA velocity, the **velocyto** Python package (La Manno et al., 2018) was utilized, which separately quantifies spliced and unspliced RNA reads. Following normalization, variable gene selection, and smoothing/imputation, the method estimates the expected steady‐state ratio between spliced and unspliced molecules using all cells in the dataset. Based on this ratio, velocyto assigns RNA velocity values for each gene in each cell to infer the future transcriptional state of the cells. The command‐line interface (CLI) of velocyto (version 0.11.0) was employed in permissive mode. Using dataset A, the data, selected the top 1000 variable genes, applied a minimum expression threshold were normalized, and performed data imputation within a neighborhood of 200 cells. RNA velocities were then calculated using the built‐in functions. Subsequently, astrocytes, RGL cells, aNSCs, pIPCs, nIPCs, and neuroblasts based on UMAP clustering (as described earlier) were isolated and their RNA velocities in UMAP space visualized. To estimate the differentiation starting points of these selected cells, a backward Markov process was applied on the transition probability matrix to identify regions of high density. All analyses were performed with default parameters unless otherwise specified.

### Ethics Approval and Consent to Participate

All animal experimental procedures in this study strictly followed the guidelines of the Institutes of Health for the Care and Use of laboratory animals and were approved by the Institutional Ethics Committee of the Second Affiliated Hospital, Zhejiang University School of Medicine (Approval no. 2022–031).

Collection and use of CSF samples was approved by the Ethics Committee of Zhejiang University (Approval no. 2023‐0315). Written informed consent was obtained from patients before the start of this study.

## Conflict of Interest

The authors declare no conflict of interest.

## Author Contributions

S.G. and X.G. contributed equally to this work and J.W. as co‐first author. Y.H. and J.W. conceived and designed the study. H.H. performed the bioinformatic analysis. S.G., X.G. and J.Z. established the CCI model, performed the flow cytometry assay and wrote the manuscript. C.Y. and S.G. analyzed the data and prepared the figures. J.L. and JJ.W. performed the neurological function evaluation. C.X., Z.Z., and N.W. performed the ILC2 adoptive transfer. JJ.W. and J.L. collected the clinical data. J.W. and S.G. performed the NSCs in vitro experiments. S.T. polished the manuscripts. All the authors have read, revised, and finally approved the manuscript.

## Supporting information



Supporting Information

## Data Availability

The data that support the findings of this study are available from the corresponding author upon reasonable request.
