# Supplementary material for: Choroid Plexus Fibroblast–ILC2 Niche Promotes Adult Hippocampal Neurogenesis after Traumatic Brain Injury
Source: Adv Sci (Weinh). 2025 Jul 3;12(35):e15984. doi: 10.1002/advs.202415984 (PMC12463046; doi:10.1002/advs.202415984)
Supplement: Supplementary file 1 — Supporting Information [file ADVS-12-e15984-s001.docx]

**Table 1. Key Resources**

| **Reagent or resources** | **Source** | **Cat #** |
| --- | --- | --- |
| **Antibodies** | | |
| Anti-CD31 Antibody | R&D Systems | AF3628 |
| Anti-PDGFRα Antibody | Thermo Fisher Scientific | 14-1401-82 |
| Anti-PDGFRα Antibody | CST | 3174 |
| Anti-AQP1 Antibody | Proteintech | 20333-1-AP |
| Anti-GATA3 Antibody | R&D Systems | MAB6330 |
| Anti-CD3 Antibody | Thermo Fisher Scientific | 14-0032-82 |
| Anti-CD45 Antibody | CST | 70257 |
| Anti-IL33 Antibody | R&D Systems | AF3626 |
| Anti-VCAM-1 Antibody | Thermo Fisher Scientific | 14-1061-82 |
| Anti-ICAM-1 Antibody | Thermo Fisher Scientific | 14-0541-82 |
| Anti-Col IV Antibody | Abcam | Ab6586 |
| Anti-CD68 Antibody | Thermo Fisher Scientific | PA578996 |
| Anti-Iba-1 Antibody | Abcam | Ab5076 |
| Anti-DCX Antibody  Anti-Ly6G antibody  Anti-CD8 antibody | CST  Proteintech  Abcam | 4604  65140-1  Ab217344 |
| Anti-GFAP Antibody | CST | 3670 |
| Anti-BrdU Antibody | Abcam | Ab6326 |
| Anti-Ki67 Antibody | Thermo Fisher Scientific | 14-5698-80 |
| Anti-SOX2 Antibody | Novus | AF2018 |
| Anti-CD45 Antibody, BV421 | Biolegend | 103155 |
| Anti-Lineage Antibody with isotype, PerCP/Cy5.5 | BD Pharmingen | 561317 |
| Anti-ICOS Antibody, APC | Biolegend | 313515 |
| Anti-CD90.2 (Thy1.2) Antibody | Biolegend | 105305 |
| Anti-CD127 Antibody, PE | Biolegend | 135009 |
| Anti-CD127 Antibody, APC | Biolegend | 135011 |
| Anti-ST2 Antibody, BV421 | Biolegend | 145309 |
| Anti-AREG Antibody, FITC | Santa cruz | sc-74501 |
| Anti-IL5 Antibody, APC | Biolegend | 153405 |
| Anti-IL13 Antibody, PE | Biolegend | 159403 |
| Anti-CD106 (VCAM-1) Antibody, FITC | Biolegend | 105705 |
| Anti-CD54 (ICAM-1) Antibody, PE-Cy7 | Biolegend | 116121 |
| Anti-PECAM-1 Antibody, PE | BD Pharmingen | 561073 |
| Anti-EpCAM Antibody, PerCP/Cy5.5 | Biolegend | 118219 |
| Anti-CD140α Antibody, APC | Biolegend | 135907 |
| Anti-CD45 Antibody, APC | Biolegend | 103112 |
| Anti-CD11b Antibody, PE-Cy7 | Thermo Fisher Scientific | 25-0112-82 |
| Anti-Ly6G Antibody, Pacific Blue | Biolegend | 127611 |
| Anti-Ly6C Antibody, BV605 | Thermo Fisher Scientific | 416-5931-82 |
| Anti-CD11c Antibody, BV510 | Biolegend | 117337 |
| Anti-MHC-11(I-A/I-E) Antibody, PE | Biolegend | 107607 |
| Anti-CD3e Antibody, FITC | BD Pharmingen | 553062 |
| Anti-CD4 Antibody, BV785 | Biolegend | 100551 |
| Anti-CD8a Antibody, PerCP/Cy5.5 | Biolegend | 100733 |
| **Recombinant proteins and chemicals** | | |
| IL33, Recombinant Protein | Biolegend | 580508 |
| IL33, Recombinant Protein | Novoprotein | CG73 |
| AREG, Recombinant Protein | R&D Systems | 989-AR |
| EGF, Recombinant Protein | PeproTech | 315-09 |
| bFGF, Recombinant Protein | PeproTech | 450-33 |
| anti-CD90.2 mAb | Biolegend | 105302 |
| StemPro Accutase | STEMCELL Tech | 07922 |
| NeuraCult proliferation medium | STEMCELL Tech | 05702 |
| **Critical commercial assays** | | |
| RNeasy Plus Micro Kit | QIAGEN | 74034 |
| Human IL-33 Quantikine ELISA Kit | R&D Systems | D3300B |
| Lineage Cell Depletion Kit | Miltenyi Biotec | 130-090-858 |
| EdU Staining Proliferation Kit | Abcam | Ab219801 |
| **Experimental animals** | | |
| Mouse: *IL33^-/-^* | GemPharmatech | T052437 |
| Mouse: *AREG^-/-^* | GemPharmatech | T027252 |
| Mouse: *EGFR^-/-^* | GemPharmatech | T052544 |


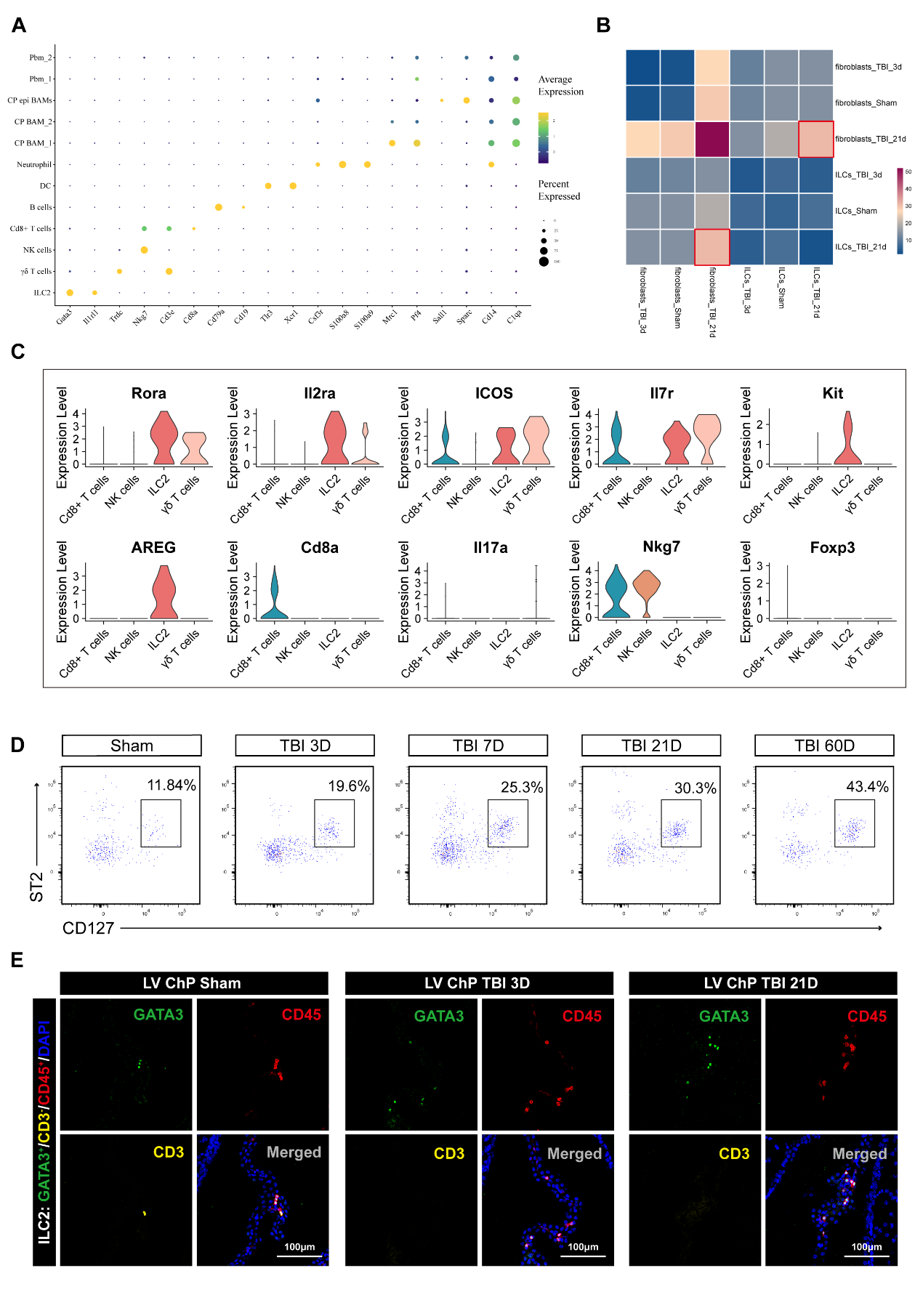


**Figure S1. Classification markers of immune sub-clusters in the choroid plexus, identification of characteristic genes and representative flow cytometry images for ILC2. Related to Figure 1.** (A) Dot plots showing the average expression (color) and the percentage of expressing cells (circle size) for characteristic marker genes (columns) in 12 sub-clusters of immune cells. (B) Heatmap showing the strength of the interaction (based on CellPhoneDB analysis) between fibroblasts and ILC2 in each group. Related to Figure 1K. (C) VlnPlot showing the expression of Rora, Il2ra, ICOS, Il7r, Kit, AREG, Cd8a, Il17a, Nkg7 and Foxp3 in 4 clusters of non-B lymphocytes. Supplement to Figure 1H. (D) Representative flow cytometry images of ILC2 after 3D, 7D, 21D, 60D of TBI and sham group. Related to Figure 1J. (E) Immunostaining of GATA3^+^ (green), CD45^+^ (red) and CD3^-^ (yellow) ILC2 in LV ChP of sham, TBI 3D and TBI 21D group mice.


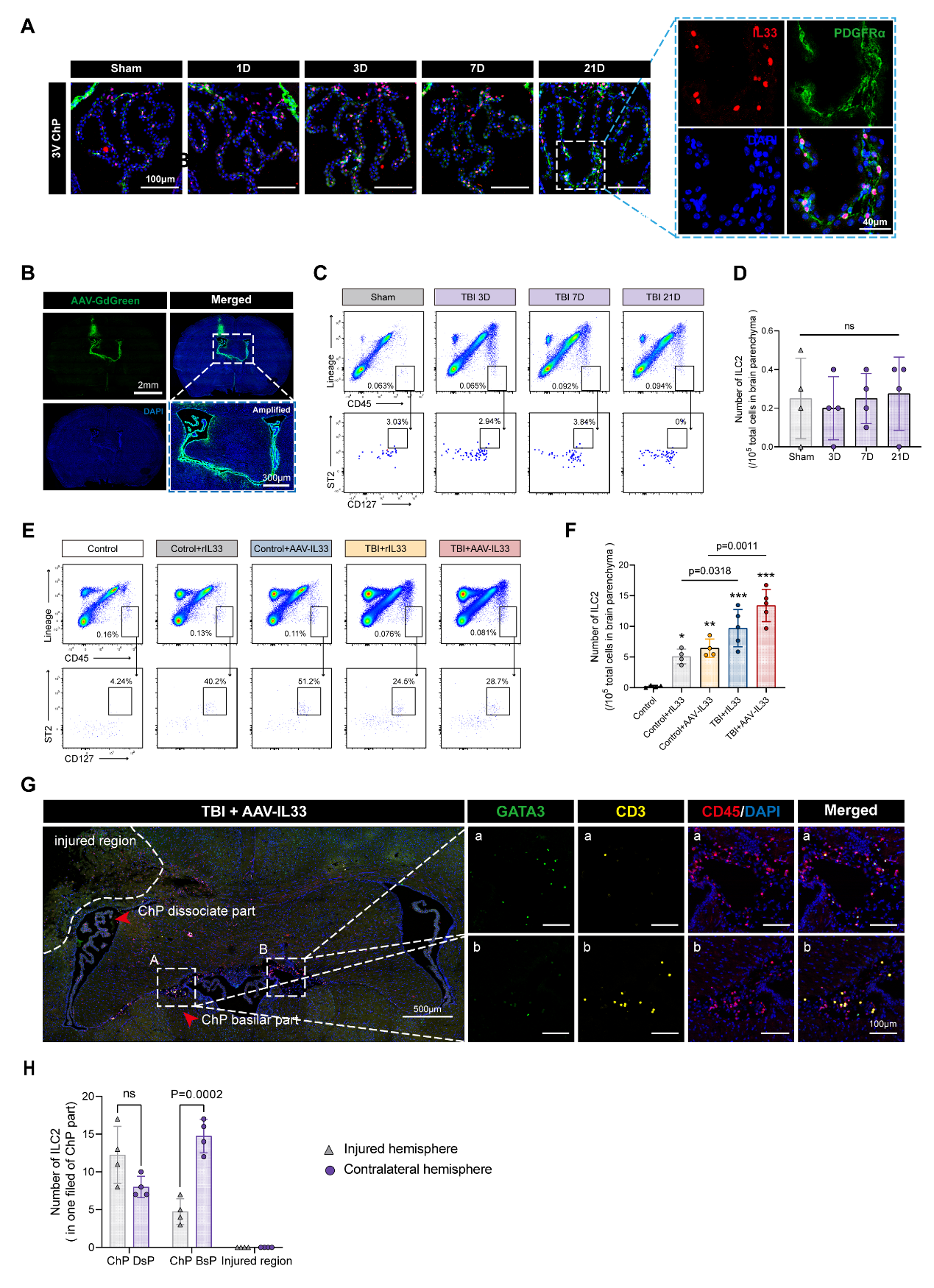


**Figure S2. ILC2 remain in the transitional zone of ChP and brain parenchyma, but not exist in the brain parenchyma, even with the treatment of rIl33 or AAV-IL33 induced overexpression. Related to Figure 2.** (A) Representative immunofluorescence images show the changes in the protein level of IL33 in the ChP of the 3V from the sham group and at different time points of TBI. The enlarged image on the right shows the co-localization of IL33 (red) and PDGFRα (green). (B) Immunofluorescence image showing specific infection of AAV2/5 on choroid plexus tissue but not the brain parenchyma. (C, D) Representative Flow cytometry pseudocolor images and quantification for ILC2 in brain parenchyma devoid of the choroid plexus after TBI. The flow scatter diagram in the second line wass enlarged for better visibility. n=4. (E, F) Representative Flow cytometry pseudocolor images and quantification for ILC2 in brain parenchyma devoid of the choroid plexus. n=4. (G) Representative immunofluorescence image showing ILC2 (GATA3^+^CD45^+^Cd3^-^) remain in the transitional zone of ChP and brain parenchyma (ChP basilar part), but not other region. (H) Quantification of Fig S2F. n=4.


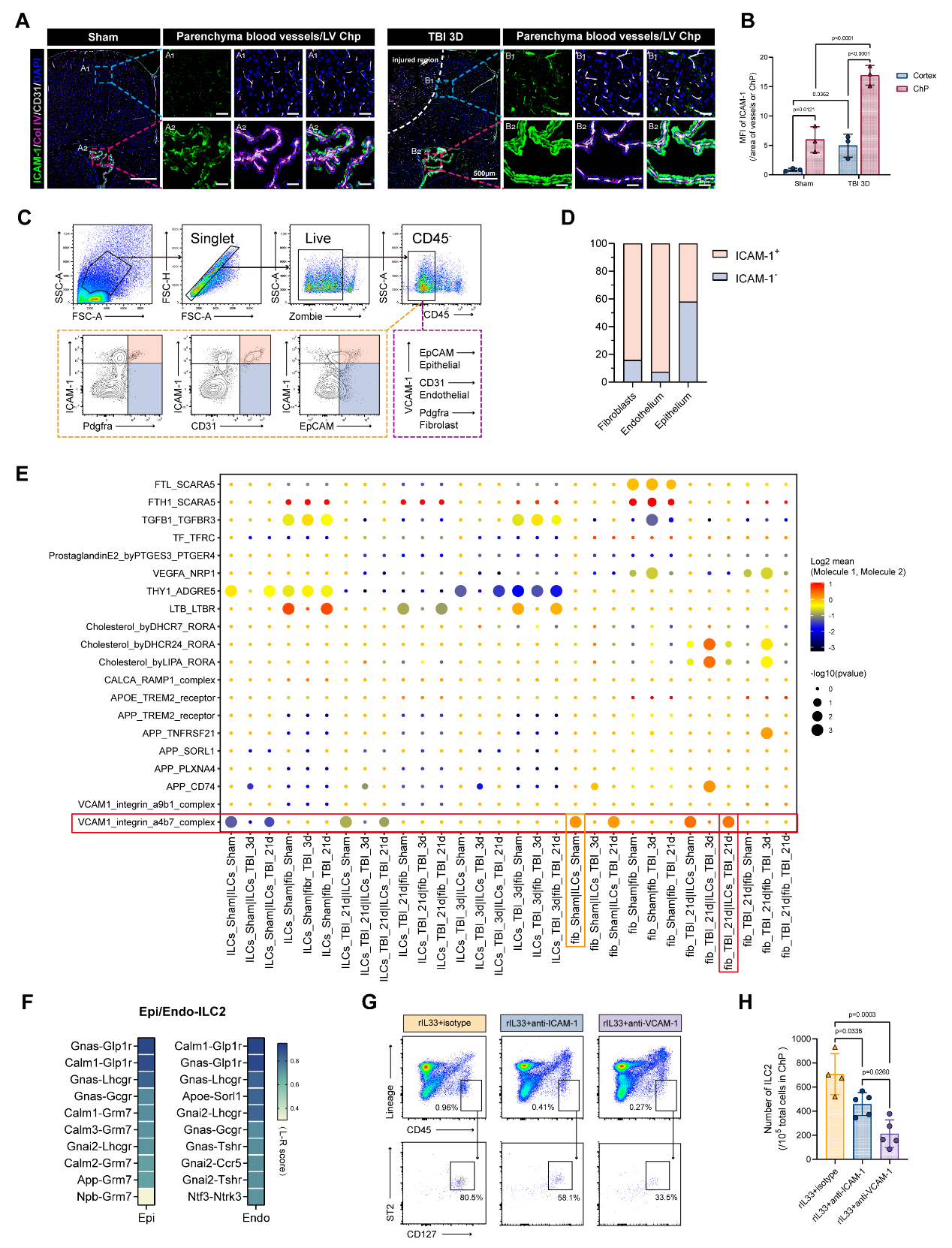


**Figure S3. The ChP ICAM-1 related interactions do not show significant recruitment effect on ILC2. Related to Figure 3.** (A, B) Immunofluorescence staining showing the expression pattern and intensity changes of ICAM-1 in ChP (red dashed frame) and brain parenchyma (blue dashed frame) after TBI. n=3. (C, D) Flow cytometry analysis the proportions of ICAM-1 expression in fibroblast (PDGFRα^+^), endothelial (CD31^+^) and epithelial (EpCAM^+^) from the naïve mouse ChP. (E) Dot plots generated by CellPhoneDB analysis showing the top 20 ligand-receptor pairs between fibroblast and ILC2 from sham, TBI 3D and TBI 21D groups. Dots are colored by interaction strength of ligand-receptor pair. (F) Heatmap showing the top 10 ligand-receptor pairs for ILC2 interacting with epithelial and endothelial, assessed by calculating the ligand‒receptor score (L-R score). (G, H) Representative Flow cytometry pseudocolor images and quantification for ILC2 in ChP. n=4, each sample was merged from 3 mice.


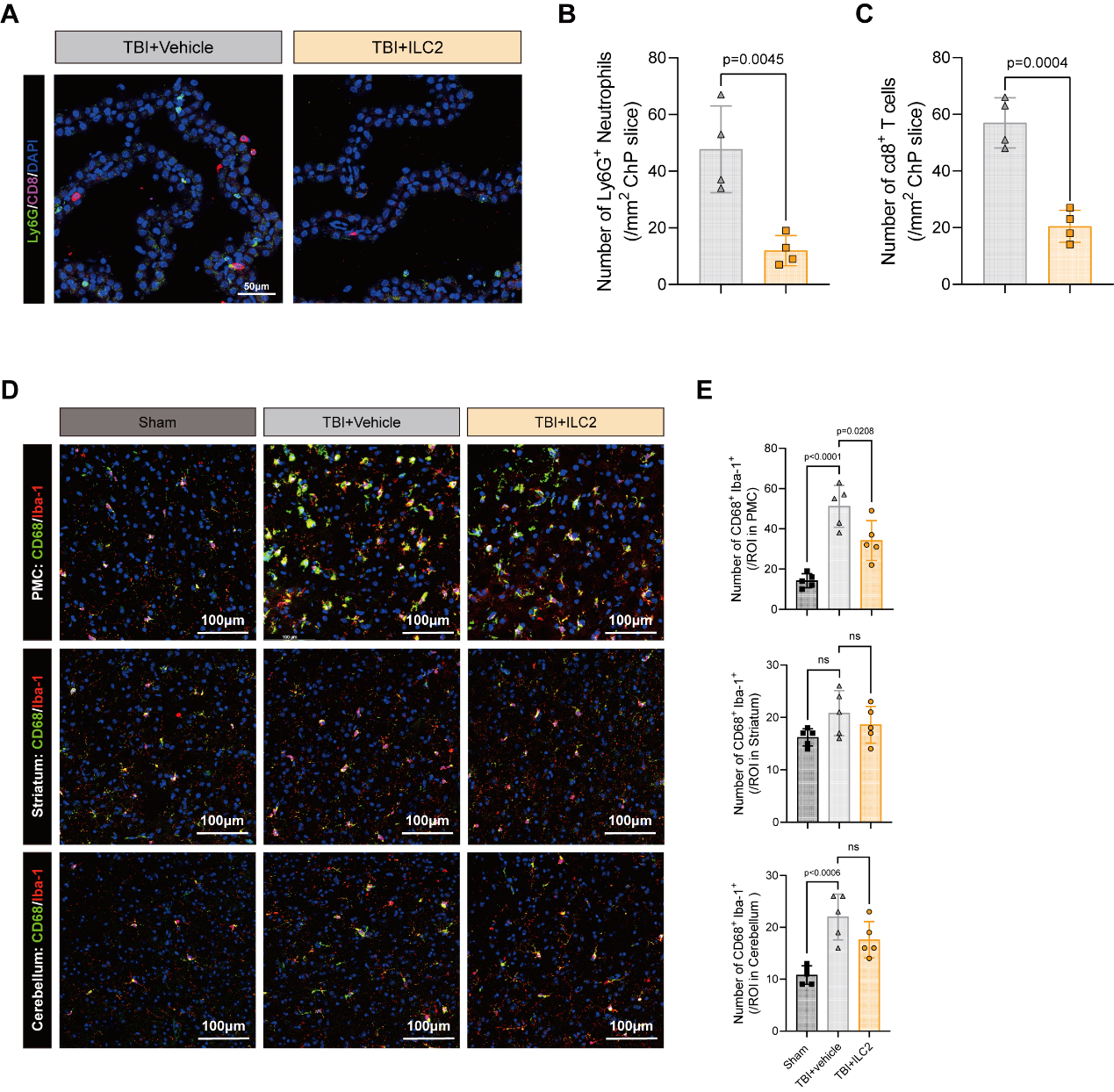


**Figure S4. Intraventricular adoptive transfer of ILC2 reduced the infiltration of CD8^+^ T cells and** **neutrophils in ChP, as well as activated microglia in premotor cortex (PMC) post-TBI. Related to Figure 4.** (A) Representative immunofluorescence image showing CD8^+^ T cells and neutrophils (Ly6G^+^) in ChP. (B, C) Quantification of Fig S4F. n=4. (D) Representative immunofluorescence image showing CD68^+^Iba-1^+^activated microglia in PMC, striatum, and cerebellum. (E) Quantification of Fig S4D. n=5.


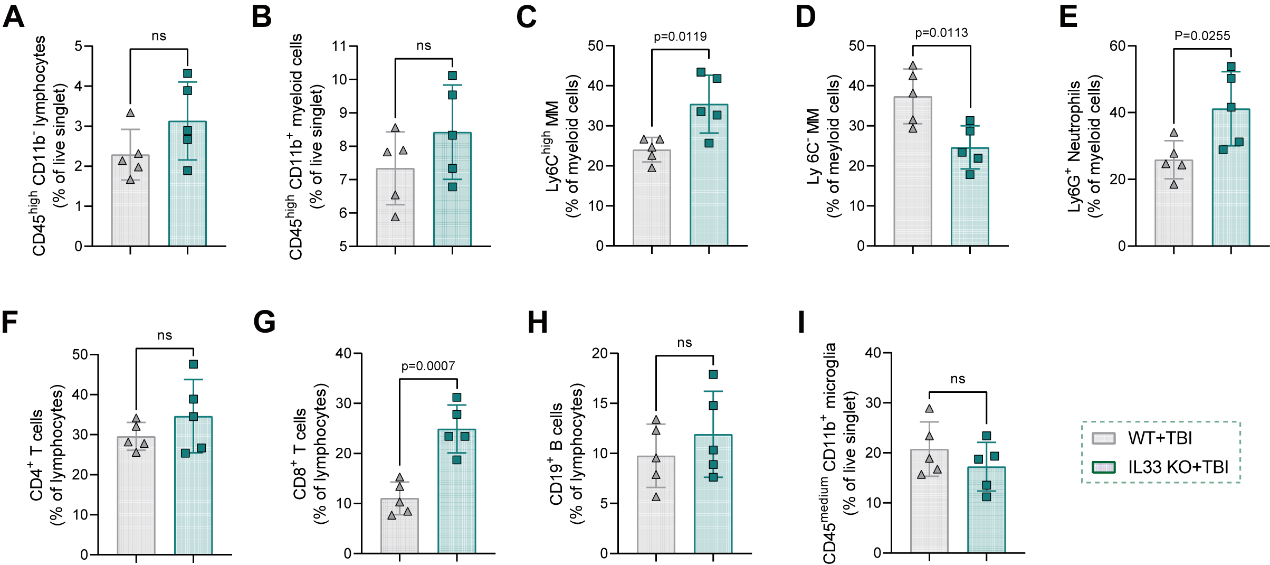


**Figure S5. IL33 KO exacerbated immune cell infiltration during the acute phase of TBI. Related to Figure 4.** (A-I) Proportion change of infiltrated immune cell in injured hemisphere from IL33 KO and WT mice group: CD45^high^CD11b^-^ lymphocyte (A), CD45^high^CD11b^+^ myeloid cell (B), Ly6C^high^ MM (C), Ly6C^-^ MM (D), Ly6G^+^ neutrophil (E), CD4^+^ T cell (F), CD8^+^ T cell (G), CD19^+^ B cell (H), CD11b^+^CD45^media^ microglia (I).


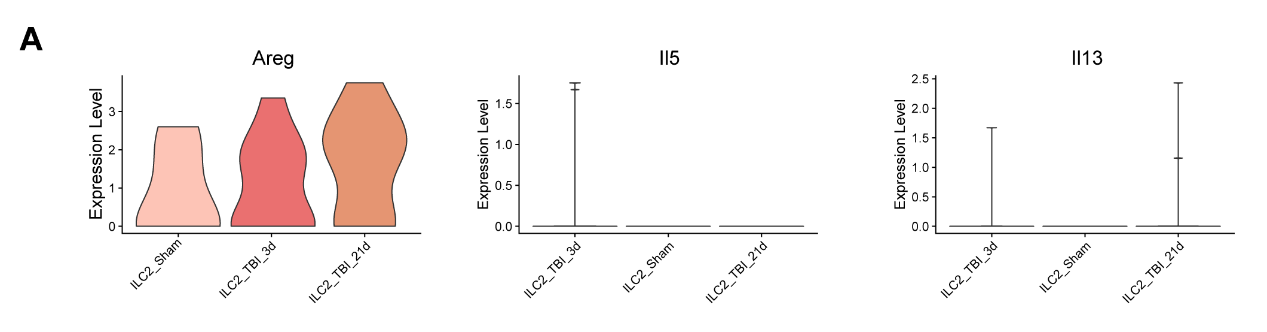


**Figure S6. The expression of AREG in ILC2 in both sham and TBI groups was significantly higher than that of IL5 and IL13. Related to Figure 6.** (A)VlnPlot showing the expression of AREG, IL5 and IL13.


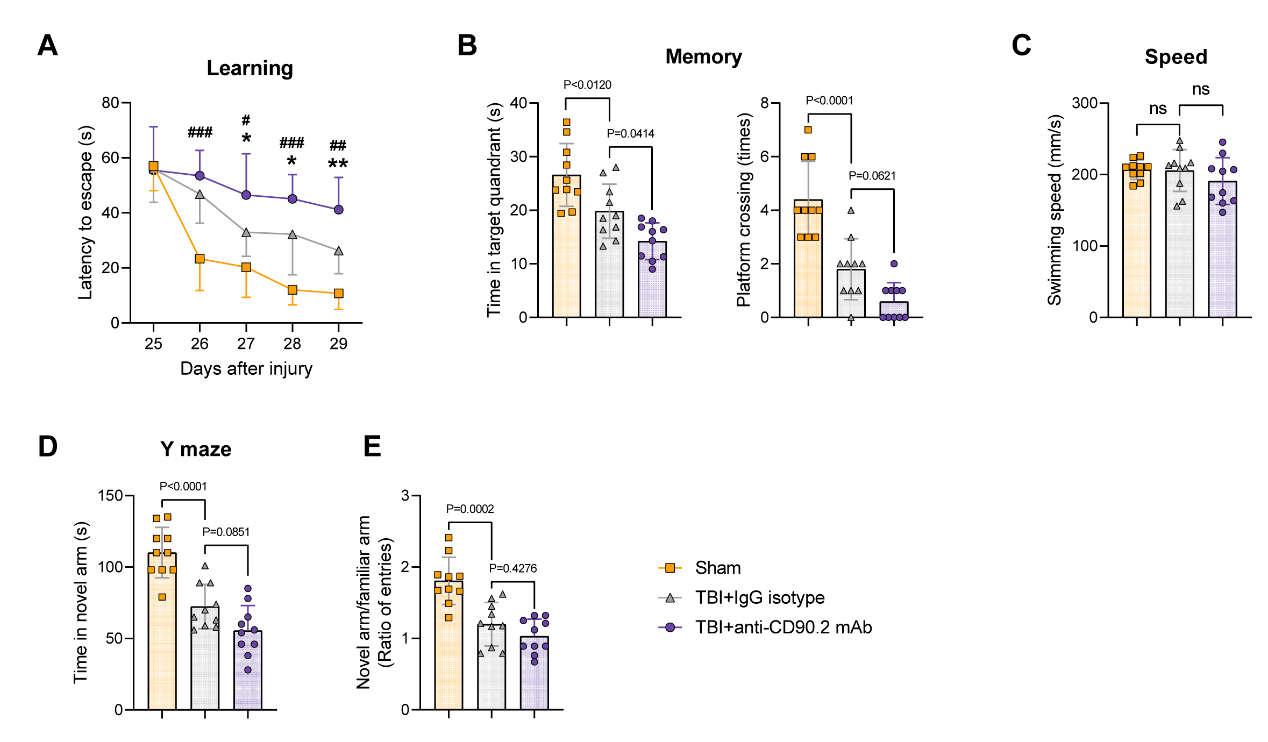


**Figure S7. Eliminating ILC2 with applying anti-CD90.2 neutralizing antibodies exacerbated memory impairment at the long term after TBI. Related to Figure 6.** (A-C) Statistical results of Morris water maze carried out in sham, IgG isotype and anti-cd90.2 mAb treated group at 25–30 days after injury. n=10. (D-E) Statistical results of new “Y maze” carried out in sham, IgG isotype and anti-cd90.2 mAb treated group at 30 days after injury. n=10.


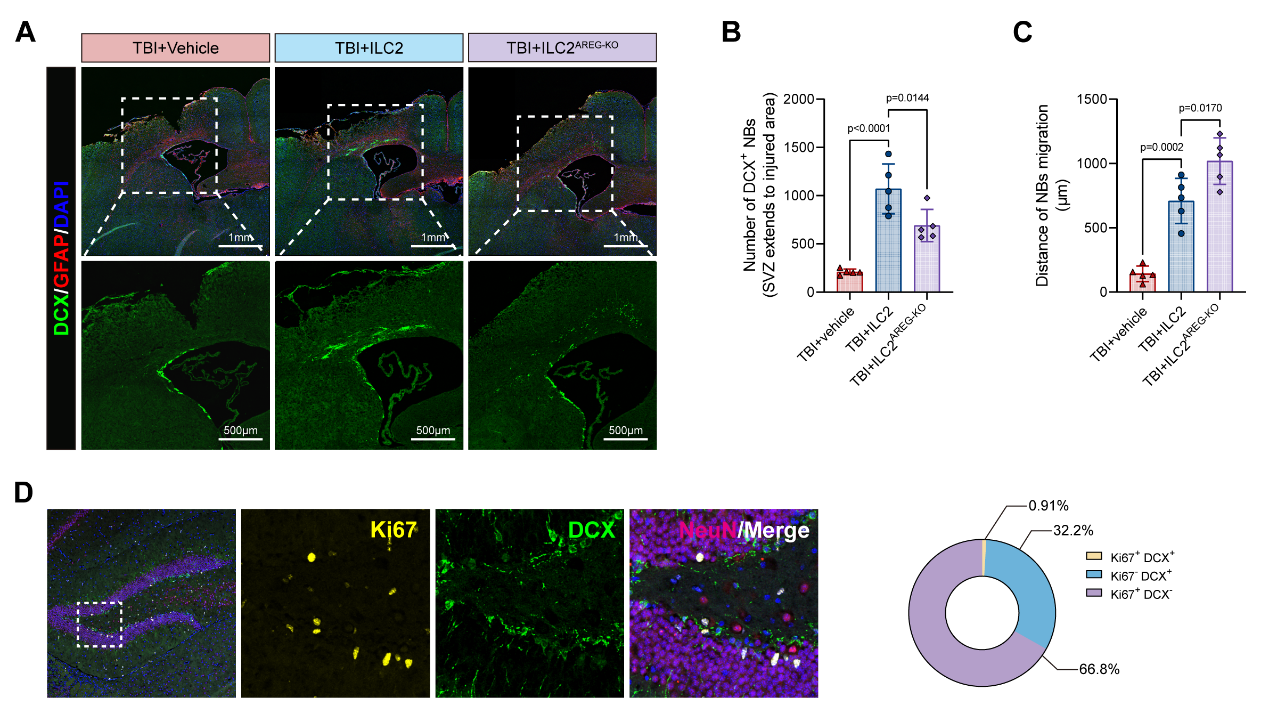


**Figure S8. ILC2 promote the amplification and migration of neuroblast originated from SVZ, but failed to induce the direct proliferation of hippocampal neuroblast after TBI. Related to Figure 5.** (A-C) Immunofluorescence images showing the quantification (B) and migration distance (C) of neuroblast (DCX^+^, green) in the coronal section of the brain from vehicle, ILC2^WT^ and ILC2^AREG-KO^ treated TBI mice. Range of neuroblast migration was enclosed by the white box in the above image were enlarged in the below image. n=5. (D) Immunofluorescence images indicating Ki67 (yellow) and DCX (green) double-positive proliferating neuroblasts, along with a pie chart showing the proportion.


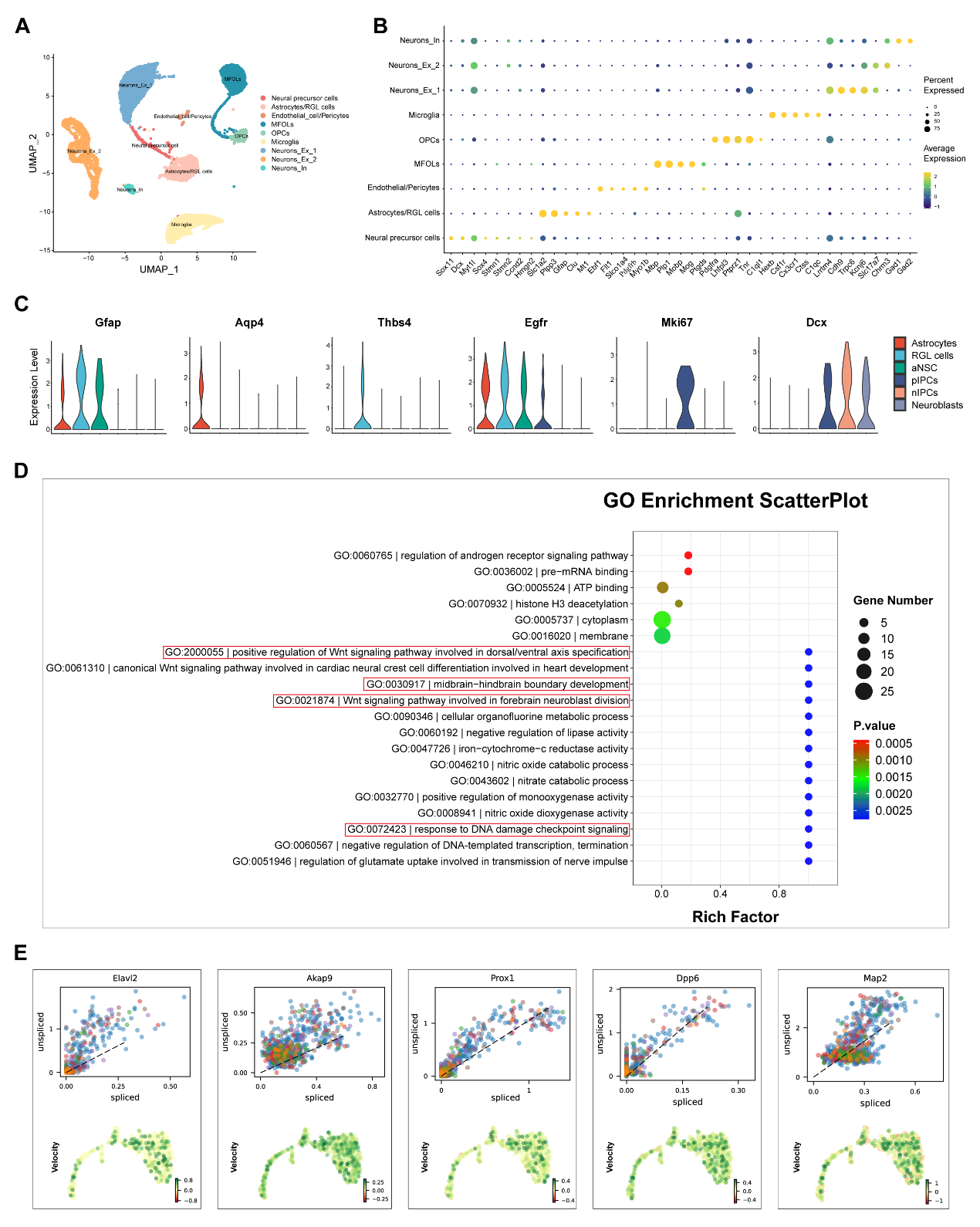


**Figure S9. Related to Figure 6.** (A, B) UMAP plots (A) and Dot plots (B) of 9 clusters for whole hippocampus from vehicle and ILC2 treated groups after TBI. (C) VlnPlot showing the dynamic changes of selected feature genes in 6 sub-clusters of neurogenic lineages. (D) GO Enrichment ScatterPlot indicating Top 20 pathways result from the comparison of qNSC from TBI_ILC2 vs TBI_Vehicle. (E) 5 of the top 6 key genes mediating RNA velocity in qNSC clusters with the treatment of ILC2 after TBI, as well as corresponding RNA velocity UMAPs.
